# Stage- and host-dependent microbiome remodeling and reciprocal changes between *Haemaphysalis longicornis* and host skin

**DOI:** 10.1128/spectrum.00864-26

**Published:** 2026-08-21

**Authors:** Nana Wei, Jinmiao Lu, Binbin Chai, Shuwen Sun, Baozhen Zhou, Zhibing Lin

**Affiliations:** 1 College of Medicine, Xi’an International University, Xi’an, China; 2 Shanghai Key Laboratory of Veterinary Biotechnology, School of Agriculture and Biology, Shanghai Jiao Tong University, Shanghai, China; 3 College of Animal Science and Technology, Inner Mongolia Minzu University 71129 , Tongliao, China; 4 College of Veterinary Medicine, Hunan Agricultural University 12575, Changsha, China; Xi'an Jiaotong-Liverpool University, Suzhou, Jiangsu Province, China; Yunnan University, Kunming, China

**Keywords:** tick, microbiome, host skin microbiome, 16S rRNA, tick-borne disease

## Abstract

Ticks harbor diverse microbial communities that are crucial for their biology and capacity to transmit pathogens. Although interactions between tick and host skin microbiomes are likely to play critical roles in feeding and pathogen transmission, these reciprocal changes during tick-host interactions remain largely unexplored. Here, we used 16S rRNA-seq to investigate how blood feeding by *Haemaphysalis longicornis* (larvae, nymphs, and adults) influences both the tick microbiome and the host skin microbiome. We further characterized microbial distribution across major tick tissues. Results revealed that feeding on different host species (mice vs rabbits) significantly altered the tick microbiome. Blood feeding reshaped microbial communities in the salivary glands and midgut, whereas the ovarian microbiome exhibited remarkable stability, suggesting the maintenance of a conserved symbiotic microbial core. Notably, *Coxiella* was identified as the dominant and stable bacterial symbiont across developmental stages and tissues and was consistently detected in eggs, suggesting that persistent vertical transmission may contribute to tick development, fitness, and nutritional homeostasis. On the other hand, *Staphylococcus* was consistently enriched at tick bite sites across host species and developmental stages, indicating that it may represent a key microbial responder involved in local microbiome remodeling and host skin responses to tick feeding. Collectively, these findings suggest that blood feeding drives dynamic remodeling of both tick and host skin-associated microbiomes and highlights *Coxiella* persistence and *Staphylococcus* enrichment as key microbial signatures of tick-host microbial interactions. Our study advances current understanding of microbiome cross-talk at the tick-host interface and provides new insights into microbiome-mediated mechanisms that may influence tick adaptation, host responses, and pathogen transmission. These findings also highlight potential opportunities for the development of microbiome-based strategies for the control of ticks and tick-borne diseases.

**IMPORTANCE:**

Microbial communities are fundamental regulators of host physiology, development, and ecological interactions. In arthropod vectors, microbiomes play important roles in development, reproduction, and pathogen transmission. However, the dynamic interactions between vector-associated microbiomes and host skin microbiomes during blood feeding remain poorly understood, particularly in ticks. Here, we found that both tick-associated and host skin microbiomes showed stage- and host-dependent alterations following tick bites. Host species differentially reshaped microbial communities across tick tissues, whereas tick bites reciprocally altered the composition of host skin microbiomes. The stable vertical maintenance of *Coxiella* and the enrichment of *Staphylococcus* at bite sites suggest that specific microbial taxa might be involved in tick and host ecological interactions. Collectively, our findings provide evidence for bidirectional microbiome modulation at the tick-host interface and highlight potential targets for the development of microbiome-based strategies to control ticks and tick-borne diseases.

## INTRODUCTION

Microbiome research has gained significant attention in recent years due to growing evidence of its profound influence on both human and animal health (1). More recently, the microbiomes of arthropod vectors and their vertebrate hosts have been increasingly recognized as important factors in vector-borne pathogen dynamics (2, 3). The skin microbiome plays a critical role in mediating interactions between blood-feeding arthropods (e.g., mosquitoes and ticks) and their vertebrate hosts. These microbial communities can influence host-seeking behavior, attachment, blood-feeding efficiency, immune modulation, and pathogen transmission (4–7). In mosquitoes, accumulating evidence has demonstrated that microbes can alter vector competence and reduce mosquito attraction to vertebrate hosts (8, 9). Despite increasing interest in both host skin microbiome and vector-associated microbiomes, their composition, interactions, and reciprocal changes during vector-host contact remain poorly understood, particularly in tick-host systems.

Ticks are obligate blood-feeding ectoparasites that transmit a wide range of pathogens, posing threats to human and animal health worldwide (2). Globally, ticks and tick-borne diseases represent a major public health concern due to their impacts on public health, animal welfare, and economic productivity (10, 11). In China, *Haemaphysalis longicornis* is a particularly important vector for more than 30 human and animal pathogens, including viruses, bacteria, and protozoa (12). As an obligate hematophagous ixodid tick with a three-host life cycle, *H. longicornis* parasitizes a wide range of host species. Although the microbiome of *H. longicornis* has been extensively investigated, most studies have focused on comparisons of field-collected ticks from different geographic regions or host species (13–15). In contrast, little is known about how host blood sources influence the microbiome of lab-reared ticks across developmental stages or how tick feeding reciprocally affects the skin microbiome of vertebrate hosts. Furthermore, the bidirectional interactions between tick-associated and host skin-associated microbial communities remain largely unexplored in a stage-specific and host-dependent context.

Here, we employed 16S rRNA-seq to characterize the microbiome profiles of lab-reared *H. longicornis* at different developmental stages following blood feeding on different hosts (mice and rabbits). We also investigated the reciprocal changes in both tick and host skin microbiomes following tick feeding using mouse and rabbit models. Our study revealed that the tick microbiome was modified by the host blood source. Additionally, the host skin microbiome at the bite site was deeply impacted by the tick bites. These findings provide new insights into microbiome interactions at the tick-host interface and improve our understanding of the ecological processes that may influence tick adaptation and pathogen transmission.

## MATERIALS AND METHODS

### Animals and ticks

BALB/c mice (8–10 weeks old) were purchased from SPF Biotechnology Co., Ltd. (Beijing, China), and New Zealand white rabbits (weighing approximately 3 kg) were purchased from JSJ Laboratory Animal Co., Ltd. (Shanghai, China). *H. longicornis* ticks (parthenogenetic strain) were obtained from a tick colony maintained at room temperature with 80% relative humidity in a dark incubator, as previously described (16).

### Collection of tick samples and host skin swabs

The back of each mouse was shaved and collared with a plastic capsule with a central drilling. Ticks were introduced into the capsule for feeding. Approximately 80–100 *H. longicornis* larvae, 30–40 nymphs, or 3–4 adults were placed on each mouse. Each mouse was maintained individually in a cage. For rabbits, the hair on the ears was shaved, and ticks were confined using ear bags. These infestation methods were performed, as previously described (16–18). Approximately 150 *H. longicornis* larvae, 100 nymphs, or 30 adults were placed on each rabbit ear. After engorgement, the whole ticks at different developmental stages (larvae, nymphs, and adults) were collected.

In addition, tissues from adult ticks, including salivary glands (SG), ovaries (OV), and midguts (MG), were dissected from the adults fed on different host species (three pool samples, 5–8 tissues/sample). Tick dissection was performed under sterile conditions using sterile blades as previously described (16). To investigate the potential vertical transmission of tick-associated microbiota, partial adult ticks fed on mice were allowed to oviposit and hatch under appropriate laboratory conditions. Eggs were collected at 0 and 14 days post-oviposition, and newly hatched larvae were also collected for microbiome analysis.

For skin microbiome sampling, skin swabs were collected from mice and rabbits at tick bite sites after all ticks had detached. Samples were obtained from the skin area enclosed by the feeding capsule in mice or the ear bag in rabbits, thereby ensuring a standardized sampling area (approximately 1.5 cm × 1.5 cm) corresponding to the region exposed to tick attachment and blood feeding. The control samples were obtained from mice or rabbits that were not infested with ticks but were subjected to the same experimental procedures. All samples were collected using sterile PBS-moistened swabs. To minimize technical variation, all sampling procedures were performed by the same investigator following an identical sampling protocol throughout the study.

### DNA extraction and 16S rRNA gene amplicon sequencing

Microbial DNA was extracted from tick samples and skin swab samples using the DNA Kit (Qiagen, Germany) according to the manufacturer’s instructions. Extracted DNA was assessed using a Thermo NanoDrop One (Thermo Fisher Scientific, USA), and then DNA libraries were constructed using a standard NEBNext Ultra II DNA Library Prep Kit for Illumina (New England Biolabs, USA). The hypervariable V3–V4 region was amplified by PCR as previously described (19) using the following primer pair: forward, ACTCCTACGGGAGGCAGCA and reverse, GGACTACHVGGGTWTCTAAT. The detection of the sequencing fragments was performed using the Illumina MiSeq platform (Magigene Co., Ltd., Guangzhou, China).

### Bioinformatics analysis

Raw reads were quality-filtered using fastp (version 0.14.1). Primer sequences were trimmed with cutadapt (version 1.14). Based on the overlap between paired-end reads, USEARCH (version 10) -fastq_mergepairs was applied to merge read pairs, discard non-conforming tags, and generate raw tags. Clean tags were imported into QIIME2 and processed using the DADA2 pipeline (q2-dada2, version 2020.11.0) for quality filtering, denoising, and amplicon sequence variant (ASV) inference at single-nucleotide resolution (100% sequence identity). Chimeric sequences were identified and removed using the consensus method implemented in DADA2 (version 2020.11.0). Non-chimeric ASVs were subsequently assigned taxonomy against the Silva database (version 132) using the corresponding pre-trained classifier at the default confidence threshold.

Taxonomic profiling and sample size adequacy were assessed using R (version 5.1.3). The community composition and taxonomic annotations were analyzed using R and KRONA software. The alpha diversity indices were calculated using USEARCH (version V10, http://www.drive5.com/usearch/) and R packages. The beta diversity was assessed by principal coordinates analysis (PCoA) based on distance matrices using the vegan package. Linear discriminant analysis effect size (LEfSe) was performed to identify the differentially abundant taxa across groups (20). Random Forest analysis was performed using the Magigene Cloud Platform. The number of decision trees (ntree) was set to 500. Feature importance was evaluated using mean decrease accuracy (MDA) indices. A random partition-based prediction strategy was applied for model construction and evaluation. Venn diagrams were generated using the online platform (https://www.genescloud.cn) to characterize the shared and unique taxa among different groups.

### Statistical analysis

LEfSe analysis illustrated the results of Welch’s *t*-test and Wilcoxon rank-sum tests on the two groups and multiple-group settings, respectively. Taxa with an LDA score >2.0 and a permutation-based FDR-adjusted *P* value <0.05 were considered significant discriminative biomarkers. Kruskal-Wallis test followed by a *post hoc* test (Dunn’s test) was used when more than two groups were compared, and *t*-test was used when two groups were compared. Correlation analysis was performed using the Spearman correlation coefficient in R packages, and the results were visualized using Genes Cloud platform. The common statistics and charts were performed using GraphPad Prism 10.1.2 (GraphPad Software, USA). A *P* value <0.05 was considered significant.

## RESULTS

### Alteration of the whole tick microbiome after feeding on mice and rabbits

**Fig 1:**
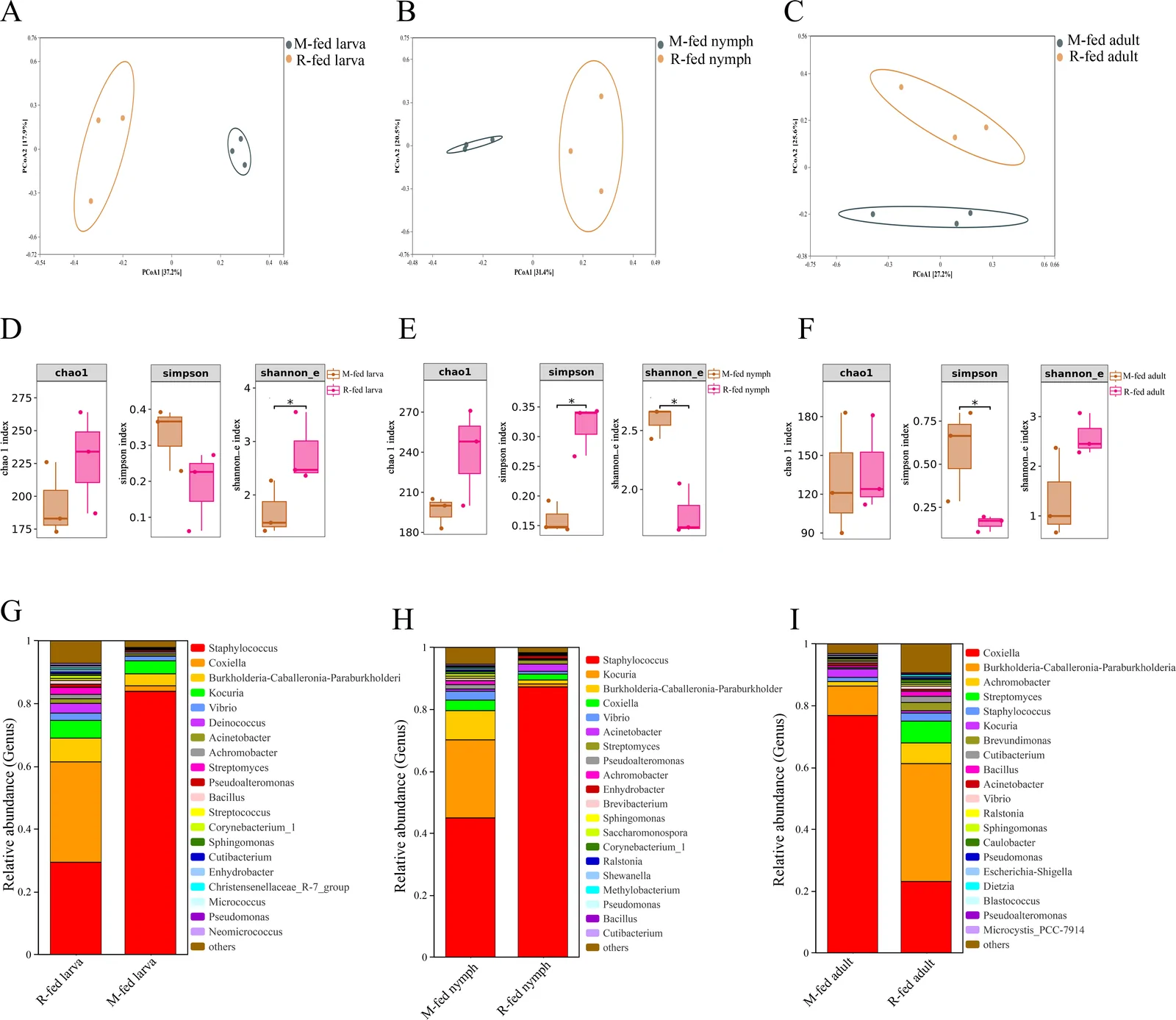
Differential alterations in bacterial microbial diversity of ticks at different developmental stages following blood feeding on different hosts. (**A–C**) PCoA of the blood-fed larva (**A**), nymph (**B**), and adult (**C**) microbiome of mouse-fed (M-fed, gray dots) and rabbit-fed (R-fed, orange dots) ticks based on unweighted UniFrac measures. (**D–F**) Alpha diversity boxplot of the fed larva (**D**), nymph (**E**), and adult (**F**) microbial community based on the Simpson, Chao 1, and Shannon_e indexes. (**G–I**) Average compositions and relative abundances of top 20 genera for fed larva (**G**), nymph (**H**), and adult (**I**).

The host blood meal source is crucial for tick development and plays an important role in the transmission of tick-borne pathogens (6). To determine how different blood meal hosts influence microbiome dynamics across tick developmental stages, we analyzed and compared the microbial communities of *H. longicornis* across developmental stages following feeding on mice vs rabbits. PCoA revealed clear separation of the microbiota between ticks fed on mice (M-fed) and those fed on rabbits (R-fed) at the larval (Fig. 1A), nymphal (Fig. 1B), and adult (Fig. 1C) stages. Regarding alpha diversity, significant differences were observed in the Shannon and Simpson indices, whereas no significant differences were detected in the Chao 1 index across the three developmental stages (Fig. 1D through F). Specifically, microbial diversity was significantly higher in M-fed nymph than in R-fed nymph (Fig. 1E), but significantly lower in M-fed larva and adult than in their corresponding R-fed groups (Fig. 1D through 1F).

We further compared the bacterial composition and relative abundance between ticks fed on mice and those fed on rabbits. Bar plots showing the relative abundance of the top 20 taxa demonstrated a dramatic impact of host blood meal source on the tick microbiome at both the phylum level (Fig. S1A through C) and genus level (Fig. 1G through I). We found that the phyla Firmicutes, Actinobacteria, and Proteobacteria together accounted for 98.79% of all sequences on average and were the three dominant bacteria in both M-fed ticks and R-fed ticks (Fig. S1A through C). However, the relative abundances of these three dominant phyla varied markedly among developmental stages within the same host group. In addition, significant differences were also observed between M-fed and R-fed ticks at the same developmental stage (Fig. S1A through C). Analysis at the genus level further confirmed the impact of host blood meal source on the tick microbiome. In larvae, *Staphylococcus* and *Coxiella* were the core bacterial genera in both M-fed and R-fed groups. Compared with R-fed larvae, M-fed larvae exhibited a significantly higher relative abundance of *Staphylococcus* and a lower relative abundance of *Coxiella* (Fig. 1G). In nymphs, *Staphylococcus* and *Kocuria* were the dominant genera, and their abundances differed significantly between M-fed and R-fed ticks (Fig. 1H). In adults, *Coxiella* and *Burkholderia-Caballeronia-Paraburkholderia* represented the predominant genera, with significant differences in their relative abundances observed between M-fed and R-fed ticks (Fig. 1I). Venn diagram revealed that 96 of 869 ASVs were shared between M-fed and R-fed larvae (Fig. S1D), 106 of 837 were shared between M-fed and R-fed nymphs (Fig. S1E), and 71 of 547 were shared between M-fed and R-fed adults (Fig. S1F).

**Fig 2:**
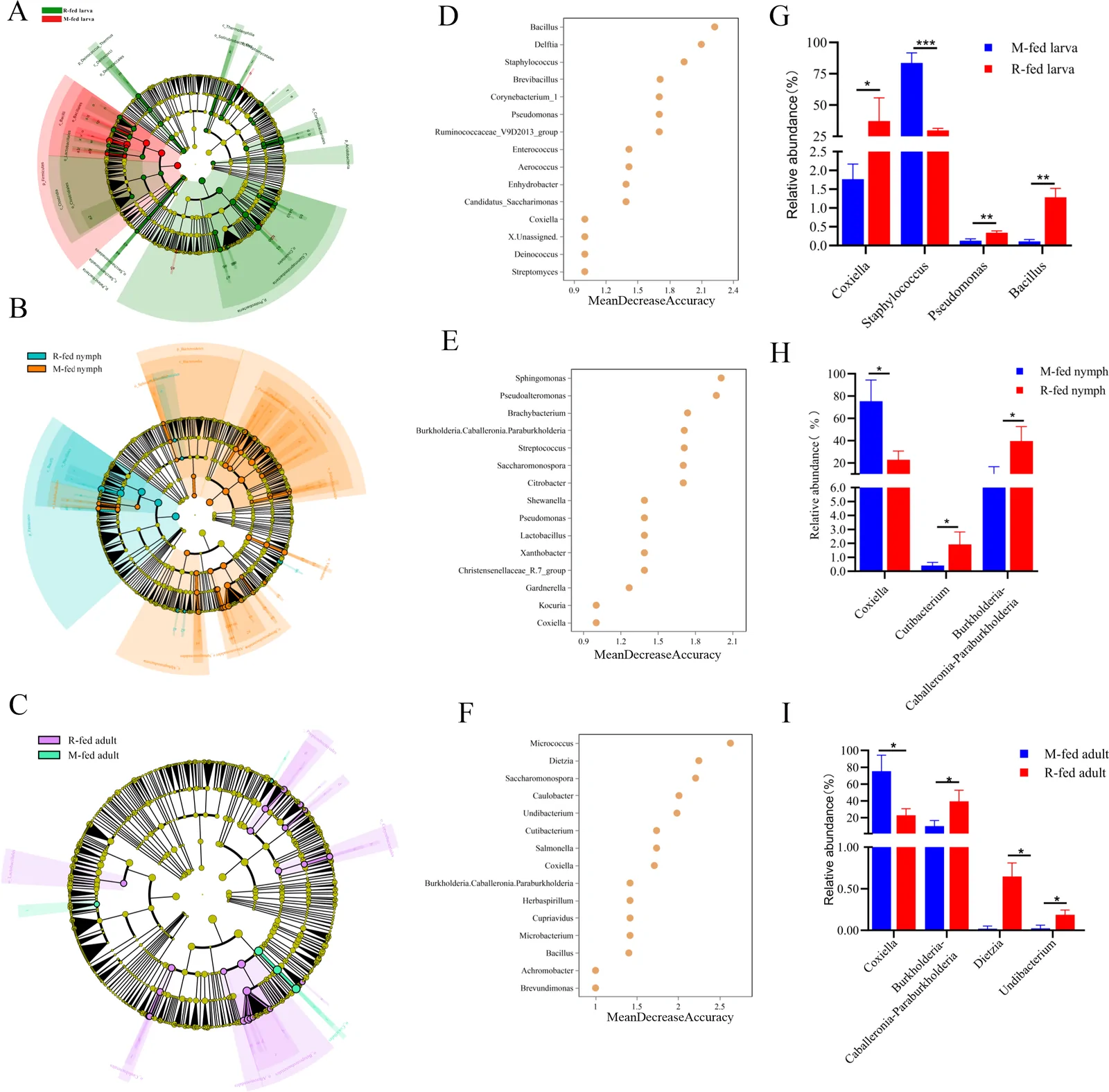
The difference and characteristics of tick microbiome in different groups. (**A–C**) LEfSe analysis of the taxa identified with a significant difference between M-fed larva and R-fed larva (**A**), between M-fed nymph and R-fed nymph (**B**), and between M-fed adult and R-fed adult (**C**), (LDA score >2.0). (**D–F**) Random Forest importance plot showing the top 15 bacterial genera that differentiate M-fed and R-fed ticks at larva (**D**), nymph (**E**), and adult (**F**). (**G– I**) Relative abundance of the bacterial genera identified as the top predictors by Random Forest analysis and showing significant differences in relative abundance (*P* < 0.05). Tick samples were compared between M-fed and R-fed ticks at the larval (**G**), nymphal (**H**), and adult (**I**) stages. *P < 0.05, **P < 0.01, and ***P < 0.001. Error bars stand for the mean ± SD.

To further characterize the variations in microbiota composition and identify specific taxonomic biomarkers between the M-fed and R-fed groups, we performed LEfSe analysis. An LDA score of >2 was used as the cut-off value. The results showed dramatic changes in the microbial community composition between M-fed and R-fed larvae (Fig. 2A and Fig. S2A), nymphs (Fig. 2B and Fig. S2B), and adults (Fig. 2C and Fig. S2C). The majority of bacterial taxa across multiple taxonomic levels were significantly enriched or depleted between the two feeding groups.

To further identify key bacterial taxa that distinguished M-fed from R-fed ticks, Random Forest analysis was performed. The top 15 bacterial genera with the highest MDA values are shown in Fig. 2D through F. Among these genera identified by Random Forest, those with significant differences in relative abundance (*P* < 0.05) are shown in Fig. 2G through I. In larval ticks, we found that those with significant relative abundance differences (*P* < 0.05) *Coxiella*, *Pseudomonas,* and *Bacillus* were more abundant in the R-fed group, while *Staphylococcus* was mainly enriched in the M-fed group (Fig. 2G). In the nymphs, M-fed ticks exhibited a higher relative abundance of *Coxiella,* but lower abundance of *Cutibacterium* and *Burkholderia-Caballeronia-Paraburkholderia* compared to the R-fed ticks (Fig. 2H). Moreover, adult tick samples from the M-fed group had a higher relative abundance of *Coxiella*, whereas they had lower abundances of *Burkholderia-Caballeronia-Paraburkholderia*, *Dietzia,* and *Undibacterium* compared to the R-fed ticks (Fig. 2I). Collectively, these findings suggested that host blood meal source significantly reshaped the composition and structure of the *H. longicornis* microbiome across developmental stages.

### Influence of host species on tissue-specific microbiome profiles of adult *H. longicornis*

**Fig 3:**
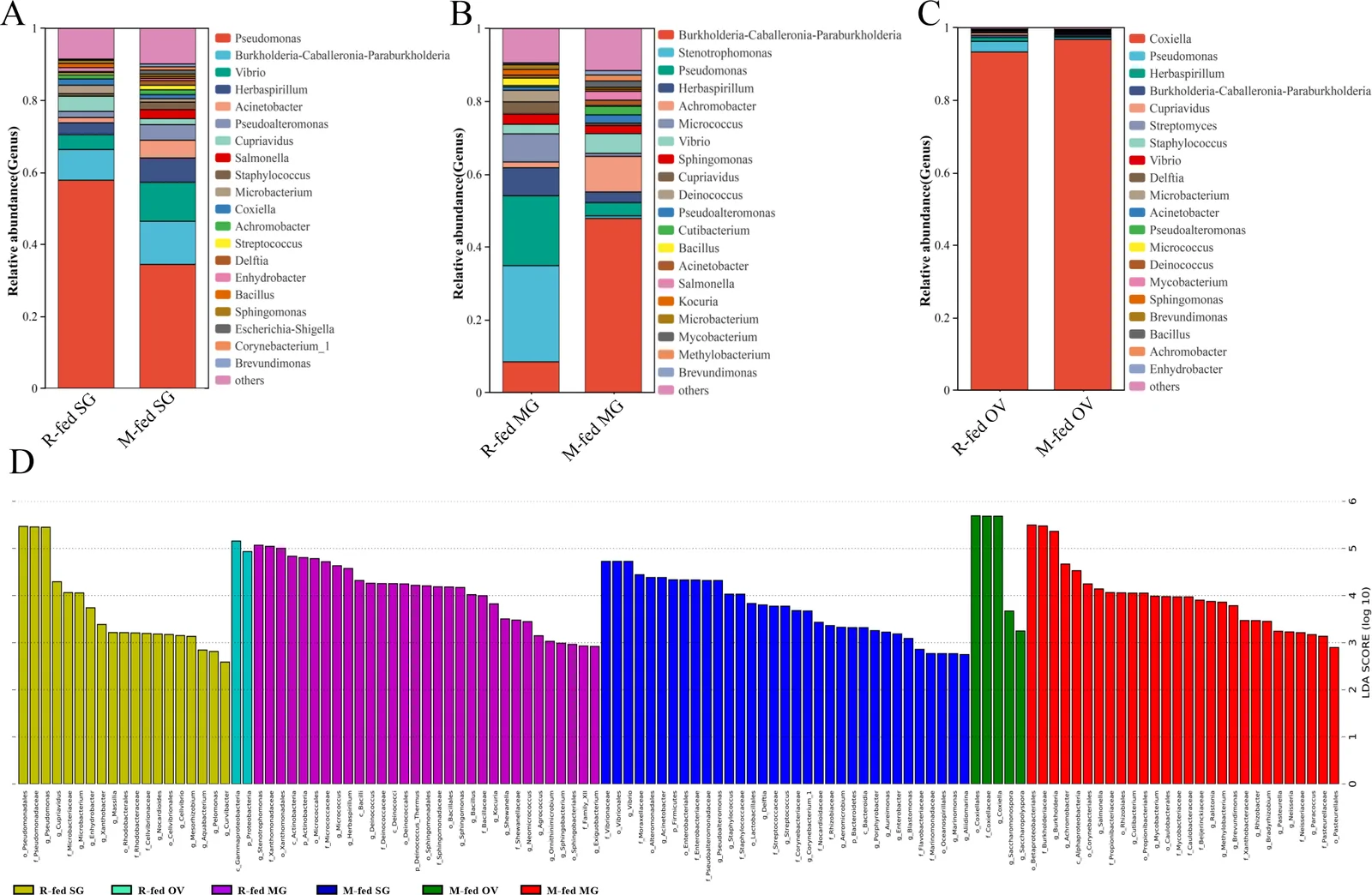
Microbial distribution in adult *H. longicornis* tissues following blood feeding on different hosts. (**A–C**) Average compositions and relative abundances of the bacterial community at the genus level in SG (**A**), MG (**B**), and OV (**C**), following feeding on mice vs rabbits. (**D**) LEfSe comparison illustrated differences in the abundant taxa with LDA score >2.0 between the six different groups.

Since feeding on different hosts markedly altered the overall tick microbiome across developmental stages, we next examined whether and how host blood meal source influenced microbial communities within specific tick tissues. Bacterial communities in the SG, MG, and OV were subsequently characterized. As shown in Fig. 3, host species exerted a greater influence on the microbial composition of the SG and MG than on that of the OV at the genus level. In SG samples, a striking decrease of *Pseudomonas* (34.42% vs 57.80%) was observed, whereas *Vibrio* (4.11% vs 10.81%) increased significantly in the M-fed group, compared to that in the R-fed group (Fig. 3A). Compared with R-fed MG samples, *Burkholderia-Caballeronia-Paraburkholderia* (47.83% vs 8.43%) and *Achromobacter* (9.79% vs 1.60%) were higher, but *Stenotrophomonas* (0.72% vs 26.43%), *Pseudomonas* (3.62% vs 19.21%), and *Herbaspirillum* (2.93% vs 7.67%) were lower in M-fed MG samples (Fig. 3B). However, the ovarian microbiota showed remarkable stability regardless of host blood meal source. *Coxiella* dominated the OV microbiota in both M-fed and R-fed ticks, accounting for 96.77% and 96.30% of the total bacterial community, respectively (Fig. 3C). LEfSe analysis confirmed the impact of host species on tissue-specific microbiome (Fig. 3D). These results indicated that blood meals from different host species induced substantial alterations in the microbiome composition of the salivary glands and midgut, whereas the ovarian microbiota remained largely unaffected.

### Vertical transmission of microbiota in ticks

**Fig 4:**
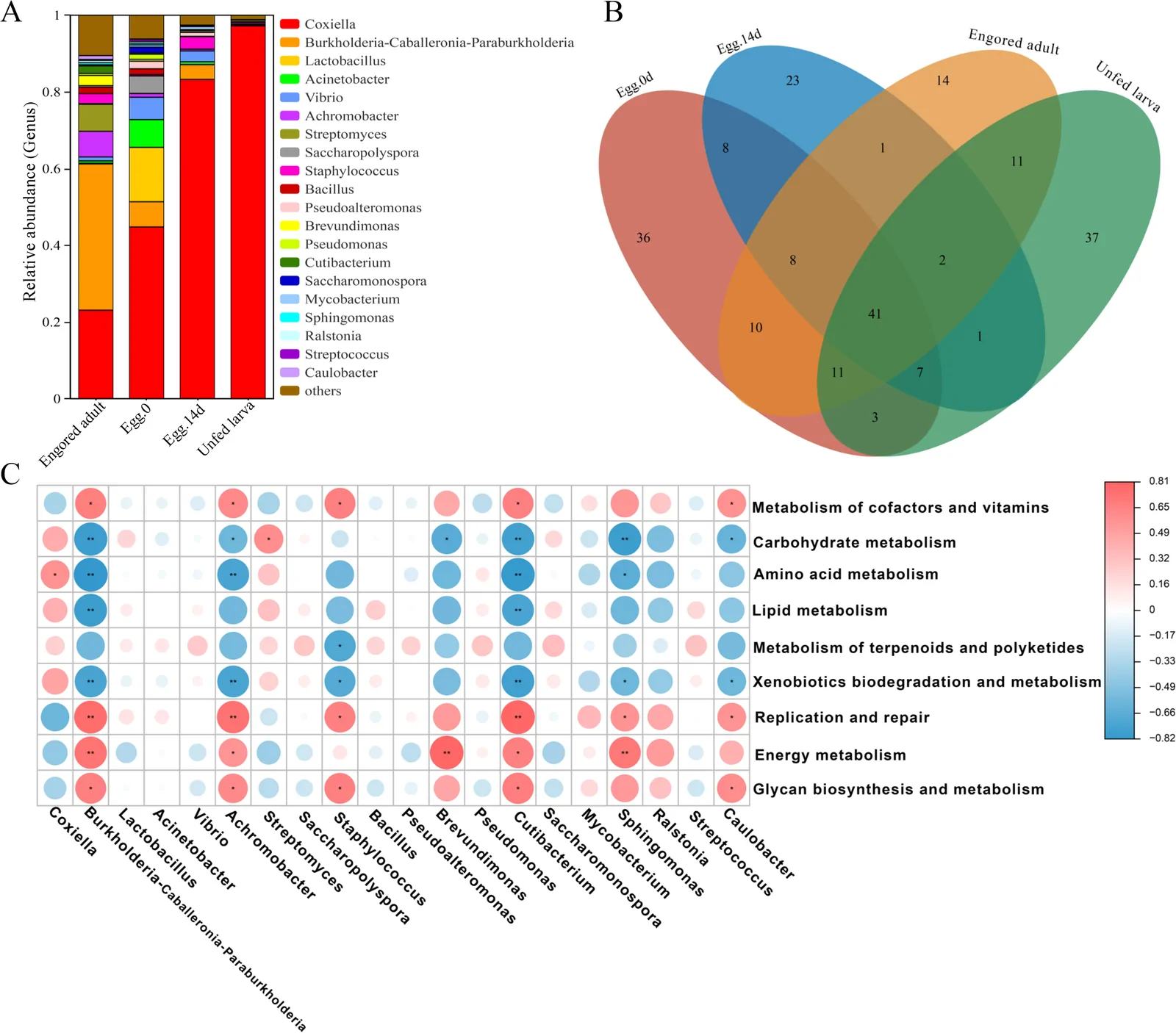
Identification of vertically transmitted microbiota via the egg in *H. longicornis* ticks. (**A**) Relative abundance of the bacterial taxa at the genus level of each group, including engorged adult ticks, eggs at 0 and 14 days, and new molted larvae. (**B**) Venn diagram depicting the shared and unique bacterial genera present in microbiomes of engorged ticks (orange), 0-day eggs (red), 14-day eggs (blue), and new molted larvae (green). (**C**) Correlation analysis of the predicted differential metabolic pathways and the top 20 genera shared across the four groups, revealing potential links between the microbiota and metabolic pathway alterations. The blue circles represent negative correlations, and the red ones represent positive correlations. The darker the color, the higher the correlation. *P < 0.05 and **P < 0.01.

The tick microbiome comprises a diverse array of symbionts which can be vertically transmitted to their offspring. These maternally inherited symbionts play vital roles in manipulating reproduction, protecting against natural enemies, or facilitating adaptation to changing environments (21, 22). To investigate the vertical dynamics of microbial transmission, we analyzed the microbiota of engorged adult ticks, newly laid eggs, and newly hatched larval ticks. As shown in Fig. 4A, the bacterial composition in engorged adult ticks (Engorged adult) was similar to that of the newly laid eggs (Egg-0d). As the ticks underwent egg laying and hatching, the relative abundance of the dominant genus *Coxiella* significantly increased, reaching 97.24% in newly hatched larvae (Fig. 4A).

Venn diagram revealed that 41 genera were shared across all four groups, whereas 37 genera were unique to newly hatched larvae (Fig. 4B). To further explore the potential functional implications of these microbial shifts, we also analyzed the correlation between the predicted differential metabolic pathways and the top 20 genera. As shown in the heatmap (Fig. 4C), nine genera showed positive correlation with four significantly changed metabolites (glycan biosynthesis and metabolism, energy metabolism, replication and repair, and metabolism of cofactors and vitamins). On the other hand, these genera showed negative correlation with five significantly changed metabolites (carbohydrate metabolism, amino acid metabolism, xenobiotics biodegradation and metabolism, lipid metabolism, and metabolism of terpenoids and polyketides). Among these analyzed genera, *Burkholderia-Caballeronia-Paraburkholderia* and *Cutibacterium* showed significant associations with eight other metabolites, with the exception of the metabolism of terpenoids and polyketides (Fig. 4C).

### Differential impacts of tick blood feeding on skin microbiomes across host species

**Fig 5:**
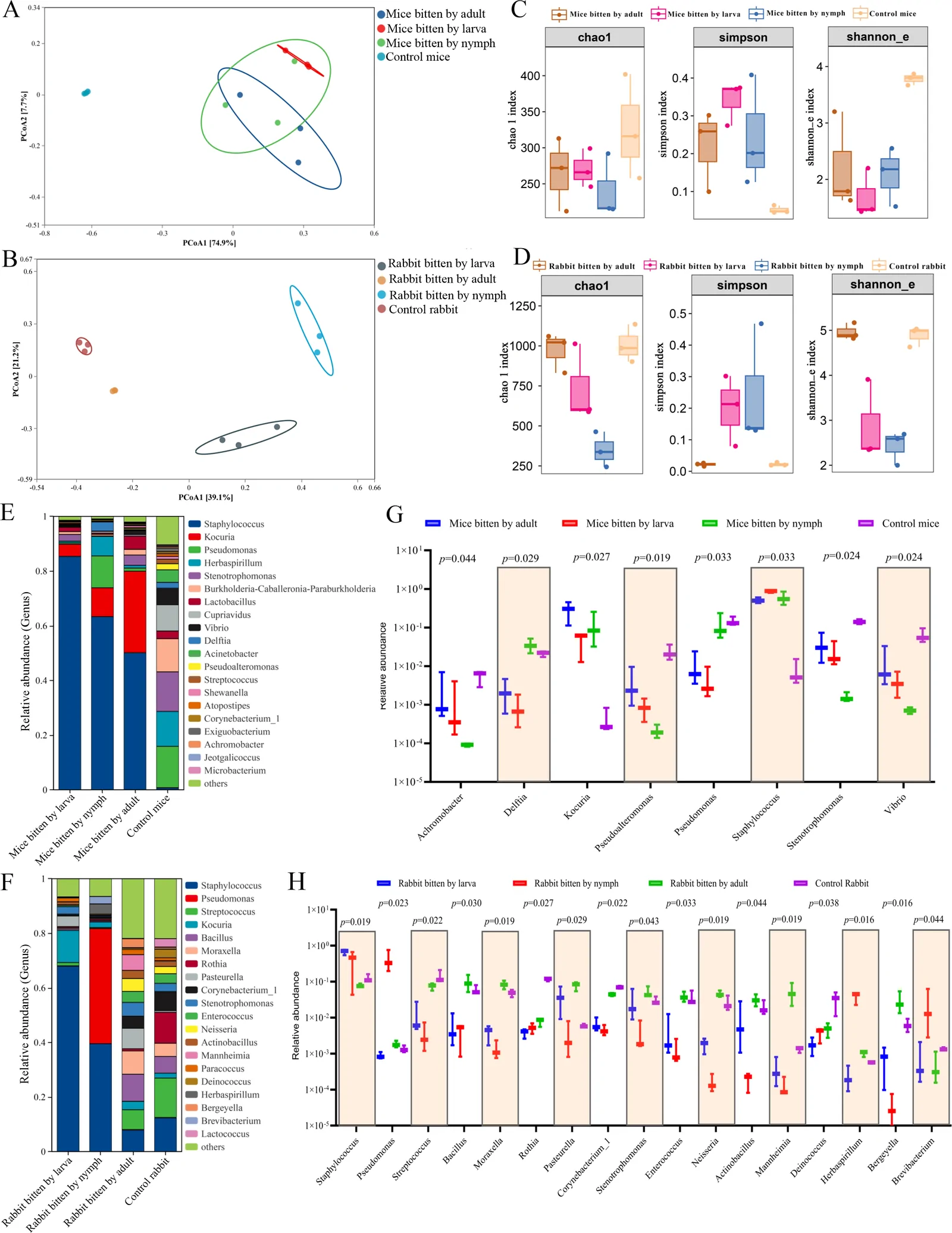
Skin microbiomes from mice or rabbit bitten by ticks at different developmental stages are deeply and differently modified. (**A**) PCoA analysis of the skin microbiome of mice bitten by larva (red dots), mice bitten by nymphs (green dots), and mice bitten by female adult ticks (dark-blue dots) and control mice (sky-blue dots). (**B**) PCoA analysis of the skin microbiome of rabbit bitten by larva (gray dots), rabbit bitten by nymphs (sky-blue dots), and rabbit bitten by female adult ticks (orange dots) and control rabbit (brick-red dots). Alpha diversity indices (boxplots of Simpson, Chao1, and Shannon_e indices) among the four groups in mouse model (**C**) and in rabbit model (**D**). Average compositions and relative abundances of the skin bacterial community in four different mouse groups (**E**) and rabbit groups (**F**) at the genus level. (**G**) Analysis of differences at the genus level among control mice, mice bitten by larva, mice bitten by nymph, and mice bitten by adult. (**H**) Analysis of differences at the genus level among four different groups in rabbit model.

Given that host species significantly influence tick microbiomes across developmental stages and tissues, we further explored whether this effect was reciprocal. Skin swab samples were collected from tick bite sites to assess the impact of tick feeding on the commensal skin microbiome of different host species. PCoA analysis showed significant differences in diversity between control mice and mice bitten by larval, nymphal, or adult ticks (Fig. 5A). And, the skin microbiota structure of rabbits bitten by larval, nymphal, or adult ticks was clearly separated from that of control rabbits (Fig. 5B). Alpha diversity analysis showed a non-significant decrease in the skin of mice bitten by larval, nymphal, or adult ticks, compared with those of control mice (Fig. 5C). As illustrated in Fig. 5D, there were no significant differences between control rabbits and rabbits bitten by adult ticks, although there was a non-significant increase between control rabbits and rabbits bitten by larvae or nymphs. Overall, tick feeding appeared to show statistically non-significant effects on alpha diversity in mouse and rabbit skin microbiota (Fig. 5C and D). In terms of bacterial composition, distinct differences were noted in the relative abundance of bacteria between tick-bitten and control skin samples (Fig. 5E through H). In mice, *Staphylococcus* comprised around 66.27% of the average relative abundance across all tick-bitten groups (Fig. 5E). A total of eight genera were differentially abundant among the four mouse groups, of which *Staphylococcus* and *Kocuria* showed higher abundance in skin bitten by larval, nymphal, or adult ticks, compared with control mouse skin (Fig. 5G). In rabbits, *Staphylococcus* (67.32%) was the predominant genus in skin bitten by larvae, whereas *Pseudomonas* (42.84%) and *Staphylococcus* (38.93%) dominated the microbiota of skin bitten by nymphs, while the top 20 genera displayed relatively uniform abundances in the skin bitten by adults (Fig. 5F). A total of 16 differentially abundant genera were identified among the four rabbit groups (Fig. 5H).

**Fig 6:**
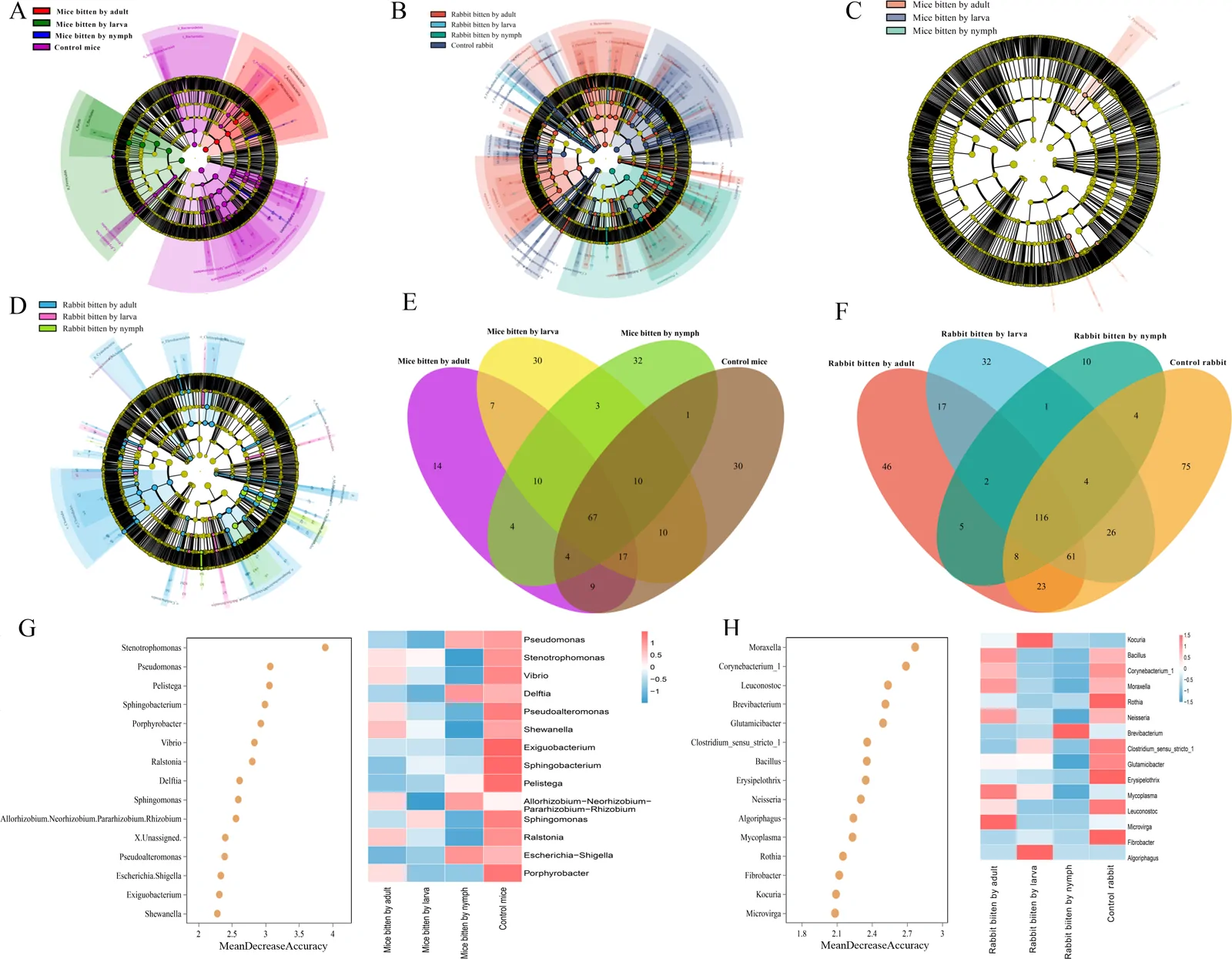
The difference and characteristics of host skin microbiome in different groups. (**A**) The LEfSe algorithm which shows the changes of the abundant taxa among control mice, mice bitten by larva, mice bitten by nymph, and mice bitten by adult. (**B**) LEfSe analysis of the taxa identified with a significant difference among four different groups in rabbit model. LEfSe analysis showed taxa with significantly different abundances among mice bitten (**C**) or rabbits bitten (**D**) by ticks of three developmental stages, respectively (LDA score >2.0). (**E**) Venn plot illustrating the unique and shared ASVs among four mouse groups. (**F**) Venn plot illustrating the unique and shared ASVs among four rabbit groups. (**G and H**) Random Forest importance plot indicating top 15 genera valuable for discriminating four groups in the mouse (**G**) and rabbit models (**H**). The importance of the genus is ordered from top to bottom, and an estimate of their importance is indicated by the corresponding MDA. Color ranging from blue to red indicates the relative abundance of different genus ranging from low to high.

As illustrated in Fig. 6, LEfSe analysis revealed remarkable differences in the microbiome composition among the four mouse groups, including control mice and mice bitten by larval, nymphal, and adult ticks. (Fig. 6A and Fig. S3A), as well as among the corresponding rabbit groups (Fig. 6B). Moreover, comparisons among tick developmental stages demonstrated that a greater number of bacterial taxa were identified as key discriminative biomarkers in rabbit skin bitten by larvae, nymphs, and adults (Fig. 6C and Fig. S3B) than in the corresponding mouse skin samples (Fig. 6D and Fig. S3C). Venn diagram showed that 67 of 248 genera were shared among all four different mouse groups, while 30, 32, and 14 genera were unique to mice bitten by larval, nymphal, and adult ticks, respectively (Fig. 6E). In rabbit skin samples, 116 of 430 genera were shared among all four groups, and 32, 10, and 46 genera were uniquely detected in rabbits bitten by larval, nymphal, and adult ticks, respectively (Fig. 6F). Furthermore, we performed Random Forest analysis to explore the potential for microbes to discriminate between control skin and tick-bitten skin. The 15 genera with the highest MDA values are shown in Fig. 6G and H. In the mouse skin samples, *Stenotrophomonas* was identified as the most important discriminative taxon and exhibited a significantly lower relative abundance in tick-bitten skin than in the skin of control mice (Fig. 6G). In the rabbit skin samples, *Moraxella* was the most important discriminative genus (Fig. 6H). Compared with control rabbit skin, *Moraxella* was enriched in skin bitten by adult ticks, but reduced in skin bitten by larval and nymphal ticks.

Collectively, these findings demonstrate that tick feeding significantly reshaped the skin microbiota of both mice and rabbits, with the magnitude and pattern of microbial alterations varying depending on host species and tick developmental stage.

### Microbial overlap and remodeling in tick and host skin microbiomes

**Fig 7:**
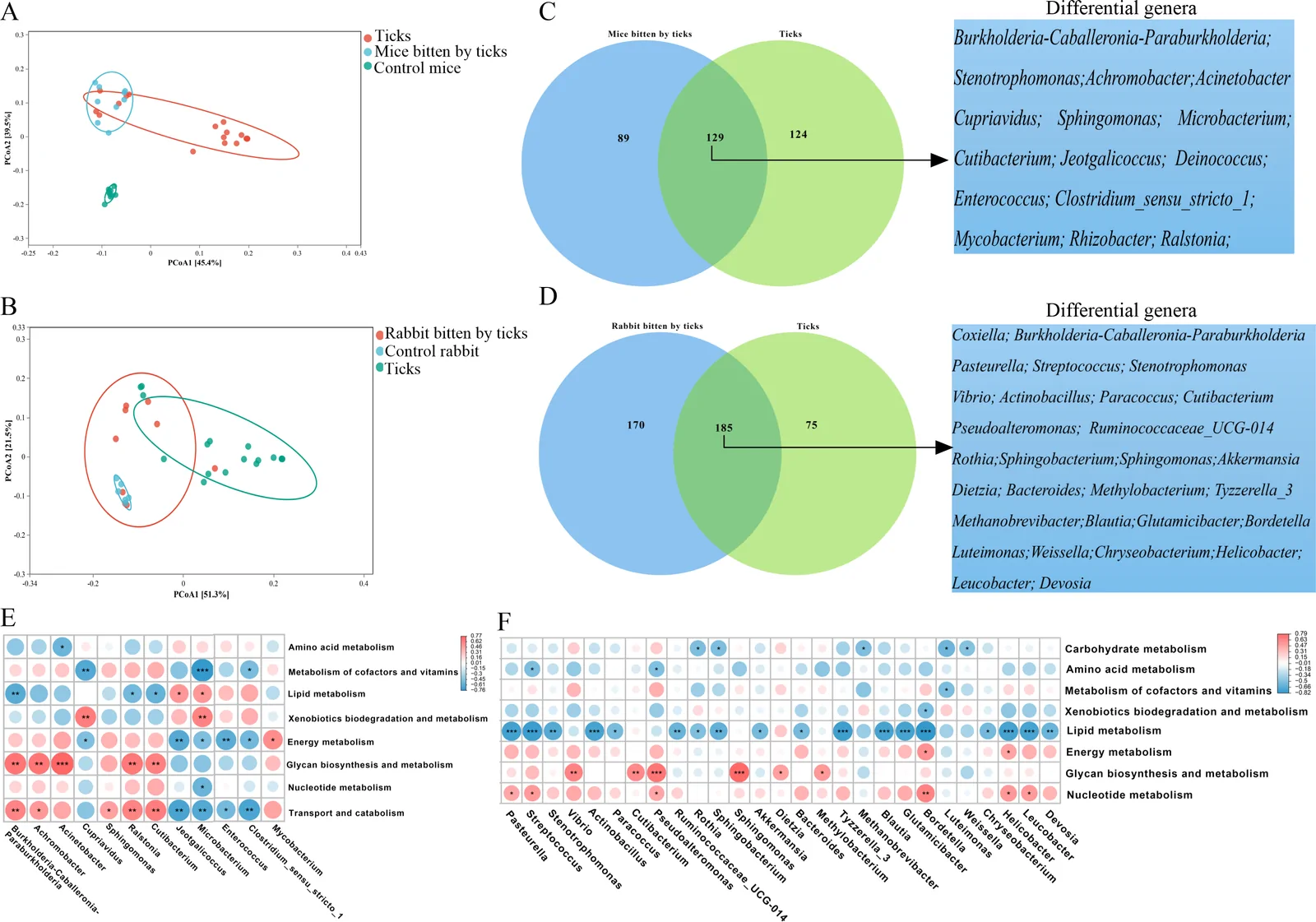
Microbial similarity and functional correlations between ticks and host skin microbiomes. (**A**) PCoA analysis at ASVs level for microbiome of the tick microbiome (all ticks, red dots) and the skin microbiome of mice bitten by ticks (blue dots) and of control mice (green dots). (**B**) PCoA comparing the tick microbiome (all ticks, green dots), the skin microbiome of rabbit bitten by ticks (red dots), and the skin microbiome of control rabbit (blue dots). (**C**) Venn diagram showing the shared and unique bacterial genera between the skin microbiomes of tick-bitten mice and all ticks fed on mice. (**D**) Venn diagram displaying the overlaps between the skin microbiomes of tick-bitten rabbit and all ticks fed on rabbit. (**E**) Correlation analysis of the shared differential genera and predicted metabolism-related pathways in the skin microbiomes of mice bitten by ticks and all mouse-fed ticks. (**F**) Correlation analysis of the shared differential genera and predicted metabolism-related pathways in the skin microbiomes of tick-bitten rabbits and all rabbit-fed ticks.

Tick infestation can induce distinct changes in the skin microbiomes of different hosts, leading to microbial overlap and potential functional remodeling. To investigate the relationship between tick microbiome and skin microbiomes of their hosts, we first performed PCoA to assess microbial community diversity. The results showed that the microbiome of tick-bitten mice was more similar to the tick microbiome (*R*² = 0.341, *P* = 0.001) than that of control mice (*R*² = 0.686, *P* = 0.001) (Fig. 7A). In rabbits, the microbiome of tick-bitten skin showed comparable levels of dissimilarity to both the tick microbiome (*R*² = 0.250, *P* = 0.001) and the control rabbit skin microbiome (*R*² = 0.233, *P* = 0.006), indicating a less pronounced microbial shift in the rabbit following tick feeding (Fig. 7B). Furthermore, Venn diagram analysis revealed 129 of 342 genera were shared between the microbiome of tick-bitten mouse skin and the tick microbiome, with 15 differentially abundant genera identified between the two groups (Fig. 7C). As shown in Fig. 7D, 185 shared genera out of 440, and 29 differentially abundant genera were identified between the microbiome of tick-bitten rabbit skin and the tick microbiome.

To evaluate the relationship between the microbial community composition and predicted metabolic functions, a correlation analysis was performed between the shared metabolic pathways predicted for the microbiomes of tick-bitten host skin and ticks, and the differential genera identified in these communities. In mice, 8 metabolic pathways were associated with 12 differentially abundant genera in the microbiomes of tick-bitten mouse skin and ticks (Fig. 7E). While 8 predicted metabolic pathways were significantly correlated with 27 differentially abundant genera in the skin microbiomes of tick-bitten rabbits and ticks (Fig. 7F). Notably, lipid metabolism exhibited significant negative correlation with most of the differentially abundant genera (Fig. 7F). In contrast, glycan biosynthesis and metabolism and nucleotide metabolism showed a positive correlation with the majority of these genera (Fig. 7F).

## DISCUSSION

Ticks are obligate hematophagous ectoparasites, and they transmit a variety of pathogens to humans and livestock during blood feeding (23). Blood feeding is a critical event in the tick life cycle and represents a major driver shaping the tick microbiome. In our present study, we found that host species significantly influenced the microbiome composition of *H. longicornis*, and these effects varied across developmental stages. Consistent with previous studies showing that host blood meal source can substantially alter tick microbial community structure (24–26), our findings further suggest that host-dependent microbiome modulation is a dynamic process throughout tick development. Several mechanisms may contribute to these host-specific effects. Differences in blood nutrient composition, immune factors, antimicrobial peptides, and other bioactive molecules among vertebrate hosts may selectively change microbial survival and proliferation of specific microorganisms within ticks. In addition, host species differ markedly in the immune responses to tick feeding, which may indirectly alter the tick-associated microbial colonization dynamics. Moreover, ticks are exposed to host skin-associated microorganisms during attachment and blood feeding, providing opportunities for microbial acquisition and exchange (27–30). Given that the skin microbiota differs substantially between mice and rabbits, exposure to distinct host skin may contribute to the differences in the microbiome observed in ticks feeding on these two host species. Together, the significant microbiome differences observed in our present study may be driven by a combination of host blood characteristics, host immune-mediated selective pressure, and interactions with host skin-associated microbiota. These findings highlight the complex ecological relationships among ticks, vertebrate hosts, and their associated microbial communities. Nevertheless, the relative contributions of these mechanisms remain unclear and require further experimental validation.

Host skin microbiota plays a unique role in maintaining skin homeostasis and regulating both innate and adaptive immune responses (31). Increasing evidence from arthropod vector studies indicates that skin-associated microbial communities play significant roles in vectors’ biology, behavior, host attractiveness, and pathogen transmission dynamics (32, 33). In mosquitoes, numerous studies have reported the microbiome profile and reciprocal changes, as well as the association between skin microbiome and the transmission of mosquito-borne pathogens, especially mosquito-borne viruses (9, 34, 35). In ticks, few studies have investigated the impact of tick feeding on the host skin microbiome. In the present study, we found that tick feeding had a substantial impact on the host skin microbiome, with significant differences observed between the two host models (mice and rabbits). Moreover, the effects of tick feeding varied greatly among the developmental stages of *H. longicornis* (larvae, nymphs, and adults), highlighting a stage-dependent influence on the host skin microbiome. After tick feeding, the skin-associated changes in mice predominantly involved key bacterial genera including *Staphylococcus*, *Pseudomonas*, and *Kocuria*, whereas in rabbits, the major changes were associated with *Staphylococcus*, *Pseudomonas*, and *Streptococcus*. The underlying mechanisms driving these host-specific and stage-dependent effects remain unclear and warrant further investigation.

Interestingly, in our present study, tick feeding-induced alterations in the skin microbiota appear to consistently involve *Staphylococcus*, suggesting that it may represent a key ecological responder in the tick-host interaction system. As a dominant and functionally important commensal of mammalian skin, *Staphylococcus* is known to respond rapidly to barrier disruption and immune modulation. In mosquitoes, an increased abundance of *Staphylococcus* has been linked to host-seeking behavior and potentially to the transmission dynamics of mosquito-borne pathogens (6, 8, 34–36). Notably, the prominence of *Staphylococcus* in our study contrasts with previous reports based on mouse skin biopsy samples, where changes were primarily observed in deeper or tissue-associated taxa (5). This discrepancy may reflect differences in sampling depth and ecological niche, as skin swabs primarily capture surface-associated microbial communities that directly interact with tick mouthparts and saliva during blood feeding. Thus, our findings highlight that *Staphylococcus* may serve as a potential early and surface-level microbial marker of tick bite-induced skin microbiome perturbation. Although associations between *Staphylococcus* and arthropod-related processes have been reported in other systems, including mosquito-host interactions, direct mechanistic evidence in ticks remains unclear. It is possible that *Staphylococcus* participates in shaping tick host-seeking behavior and influencing pathogen transmission dynamics; however, these speculations require direct experimental validation in future studies.

The stable vertical transmission of *Coxiella* suggests that specific symbiotic bacteria form a conserved core microbiome that supports tick physiology. The enrichment of *Staphylococcus* at tick bite sites indicates localized ecological interactions between host skin microbes and tick feeding activity. Together, these findings highlight a bidirectional microbiome interaction between ticks and hosts, forming a tripartite ecological network involving host, tick, and their associated microbial communities.

Several limitations of this study should be acknowledged. Although clear reciprocal changes between tick and host skin microbiomes across different host species were observed, their implications for pathogen transmission were not directly assessed and warrant further investigation. Our current results were limited to the host skin surface-associated microbiota and did not capture deeper skin communities, which may respond differently to tick feeding. Finally, skin microbial changes in non-bite areas adjacent to tick attachment sites were not examined and require our future investigations. Despite these limitations, our study provides a novel and integrative profile of stage- and host-dependent reciprocal changes between tick and host skin microbiomes.

### Conclusions

In conclusion, our study demonstrates that blood feeding by *H. longicornis* induces marked stage- and host-dependent alterations in both tick-associated microbiomes and host skin microbiomes. Host blood meal source differentially reshaped the tick microbiome across developmental stages and tissues, while tick feeding reciprocally alters host skin microbial communities, underscoring a dynamic cross-talk at the tick-host skin interface. Notably, the significant enrichment of *Staphylococcus* on tick-bitten skin and the vertical dominance of *Coxiella* highlight the potential importance of specific microbial taxa in mediating tick-host interactions. The functional implications of microbiome cross-talk for tick-borne pathogen transmission remain to be further explored. Targeted manipulation of tick or host skin microbiomes may represent a promising strategy for the biological control of ticks and tick-borne diseases.

## Supplementary Material

Reviewer comments

## Data Availability

The sequencing data have been deposited in the Sequence Read Archive (SRA), under the accession number PRJNA1398036. The original contributions presented in the study are included in the article; further inquiries can be directed to the corresponding authors.

## SUPPLEMENTAL MATERIAL

Supplemental figure legends spectrum.00864-26-s0001.docx Figure S1: Differential alterations in bacterial microbial diversity of ticks at different developmental stages following blood-feeding on different hosts. (A-C) Average compositions and relative abundances of Top 20 most abundant phyla for fed larva (A), nymph (B) and adult (C). (D-F) Venn diagram showing the shared and unique ASVs between M-fed larva and R-fed larva (D), between M-fed nymph and R-fed nymph (E), between M-fed adult and R-fed adult (F). Figure S2: LEfSe analysis between different groups. LDA score histograms (LDA score > 2.0) showing differential taxa between M-fed and R-fed ticks at larval (A), nymphal (B), and adult (C) stages. Figure S3: LEfSe analysis among different groups. LDA score histograms showing differentially abundant taxa (LDA score > 2.0) among control and tick-bitten mice (A), mice bitten by ticks at different developmental stages (B), and rabbits bitten by ticks at different developmental stages (C).

Figure S1 spectrum.00864-26-s0002.tif Differential alterations in bacterial microbial diversity of ticks at different developmental stages following blood-feeding on different hosts.

Figure S2 spectrum.00864-26-s0003.tif LEfSe analysis between different groups.

Figure S3 spectrum.00864-26-s0004.tif LEfSe analysis among different groups.

OPEN PEER REVIEW reviewer-comments.pdf An accounting of the reviewer comments and feedback.

ASM does not own the copyrights to Supplemental Material that may be linked to, or accessed through, an article. The authors have granted ASM a non-exclusive, world-wide license to publish the Supplemental Material files. Please contact the corresponding author directly for reuse.
