# Supplementary material for: Stage- and host-dependent microbiome remodeling and reciprocal changes between Haemaphysalis longicornis and host skin
Source: Microbiol Spectr. 2026 Aug 21;14(10):e00864-26. doi: 10.1128/spectrum.00864-26 (PMC13689275; doi:10.1128/spectrum.00864-26)
Supplement: Supplementary file 1 [file reviewer-comments.pdf]

ASM | Microbiology Spectrum

Stage- and host-dependent microbiome remodeling and reciprocal changes between Haemaphysalis longicornis and host skin

nana Wei, jinmiao lu, binbin chai, shuwen sun, baozhen zhou, and Zhibing Lin

Corresponding Author(s): Zhibing Lin, Xi'an International University

**Review Timeline:**		
	Submission Date:	March 10, 2026
	Editorial Decision:	May 18, 2026
	Revision Received:	June 21, 2026
	Editorial Decision:	July 5, 2026
	Revision Received:	July 23, 2026
	Accepted:	July 29, 2026

Editor: Xinzhao Tong

Reviewer(s): Disclosure of reviewer identity is with reference to reviewer comments included in decision letter(s). The following individuals involved in review of your submission have agreed to reveal their identity: Yunjian Xu (Reviewer #2)

Transaction Report:

(Note: With the exception of the correction of typographical or spelling errors that could be a source of ambiguity, letters and reports are not edited. The original formatting of letters and referee reports may not be reflected in this compilation.)

DOI: https://doi.org/10.1128/spectrum.00864-26

Re: Spectrum00864-26 (**Stage- and host-dependent microbiome remodeling and cross-alterations between *Haemaphysalis longicornis* and host skin**)

Dear Dr. zhibing lin:

Thank you for the privilege of reviewing your work. Below you will find my comments, instructions from the Spectrum editorial office, and the reviewer comments.

Please return the manuscript within 60 days; if you cannot complete the modification within this time period, please contact me. If you do not wish to modify the manuscript and prefer to submit it to another journal, notify me immediately so that the manuscript may be formally withdrawn from consideration by Spectrum.

Revision Guidelines
To submit your modified manuscript, log into the submission site at https://spectrum.msubmit.net/cgi-bin/main.plex. Go to Author Tasks and click the appropriate manuscript title to begin. The information you entered when you first submitted the paper will be displayed; update this as necessary. Note the following requirements:

• Upload point-by-point responses to the issues raised by the reviewers in a file named "Response to Reviewers," NOT in your cover letter.
• Upload a compare copy of the manuscript (without figures) as a "Marked-Up Manuscript" file.
• Upload a clean .DOC/.DOCX version of the revised manuscript and remove the previous version.
• Each figure must be uploaded as a separate, editable, high-resolution file (TIFF or EPS preferred), and any multipanel figures must be assembled into one file.
• Any supplemental material intended for posting by ASM should be uploaded with their legends separate from the main manuscript. You can combine all supplemental material into one file (preferred) or split it into a maximum of 10 files with all associated legends included.

For complete guidelines on revision requirements, see our Submission and Review Process webpage. Submission of a paper that does not conform to guidelines may delay acceptance of your manuscript.

Data availability: ASM policy requires that data be available to the public upon online posting of the article, so please verify all links to sequence records, if present, and make sure that each number retrieves the full record of the data. If a new accession number is not linked or a link is broken, provide Spectrum production staff with the correct URL for the record. If the accession numbers for new data are not publicly accessible before the expected online posting of the article, publication may be delayed; please contact production staff (Spectrum@asmusa.org) immediately with the expected release date.

Publication Fees: For information on publication fees and which article types are subject to charges, visit our website. If your manuscript is accepted for publication and any fees apply, you will be contacted separately about payment during the production process; please follow the instructions in that e-mail. Arrangements for payment must be made before your article is published.

ASM Membership: Corresponding authors may join or renew ASM membership to obtain discounts on publication fees. Need to upgrade your membership level? Please contact Customer Service at Service@asmusa.org.

The ASM Journals program strives for constant improvement in our submission and publication process. Please tell us how we can improve your experience by taking this quick Author Survey.

Thank you for submitting your paper to Spectrum.

Sincerely,
Xinzhao Tong
Editor
Microbiology Spectrum

Reviewer #1 (Comments for the Author):

This study provides a comprehensive and well executed analysis of the stage dependent and host dependent remodeling of both tick and host skin microbiomes during feeding of Haemaphysalis longicornis. The authors present compelling evidence that host species and tick developmental stages shape microbial communities, and they highlight the stable vertical transmission of Coxiella and the enrichment of Staphylococcus at bite sites. These findings offer valuable insights into tick host microbial

interactions and potential targets for biological control. However, the manuscript would benefit from several improvements.

Comments:
1. The authors have convincingly demonstrated that the microbiome of Haemaphysalis longicornis differs significantly between ticks fed on mice versus those fed on rabbits. However, the manuscript does not explore the potential reasons underlying these host dependent differences. I recommend that the authors provide a more in-depth discussion of possible causal factors, such as variations in host blood composition (e.g., nutrients, antibodies, antimicrobial peptides), differential immune pressures exerted by mice and rabbits, or the acquisition of distinct skin associated bacteria during feeding. Addressing these possibilities would strengthen the mechanistic interpretation of the findings and enhance the biological significance of the study.
2. In the Materials and Methods section, the 16S rRNA amplification targets the V3 V4 region, the primer sequences (forward and reverse) are not provided. It would be better to include this information.
3. Figures 2A C and 6A D (LEfSe cladograms). These circular cladograms contain dense taxonomic labels, but the font size and resolution are too low to be legible across all seven panels. No supplementary table lists the differentially abundant taxa (LDA > 2.0, p < 0.05) with their enrichment directions. I recommend providing enlarged, high resolution versions as supplementary figures or, preferably, a summary table that clearly presents all significant taxa for each comparison, including taxonomic level, LDA value, p value, and enrichment group.
4. Figures 2E and 2H (nymph stage), and also relevant to 2D 2G and 2F 2I. The Random Forest analysis (Figure 2E) lists the top 15 bacterial genera that discriminate M fed from R fed nymphs, yet only a subset of these (e.g., Coxiella, Cutibacterium, Burkholderia Caballeronia Paraburkholderia) are shown in the relative abundance plot (Figure 2H) with statistical significance indicators. The remaining 12 genera are not presented in Figure 2H, and neither the legend nor the main text explains whether they failed to reach significance in univariate tests. This creates ambiguity for readers who might assume that all top ranked genera from the multivariate model show significant abundance differences. I recommend that the authors clarify in the figure legend or Results section that only those genera with both high importance in Random Forest and significant univariate differences (p < 0.05) are displayed in Figures 2G, 2H, and 2I. A brief statement, for example "Among the top 15 genera identified by Random Forest, those with significant relative abundance differences (p < 0.05) are shown in G I", would greatly improve transparency.
5. In the Discussion section, the authors frequently restate detailed findings from the Results, which reduces clarity and impact. I suggest substantially condensing this redundancy and instead focusing on two aspects: first, interpreting the biological and ecological implications of the findings; second, addressing the practical significance more clearly. Specifically, at each stage of tick-host interaction, the tick microbiome and host skin microbiome influence each other during coevolution. Understanding this mutual influence can help identify potential measures to protect hosts, including developing new control strategies, drugs, or vaccines. A clearer discussion of this application value would help readers appreciate the study's relevance to the biological control of ticks and tick-borne diseases.
6. The manuscript presents interesting findings, but the writing requires revision to improve clarity, grammar, and professional tone. Several issues were noted throughout the text. First, there are grammatical errors and incorrect word choices. For example, in the Introduction: "H. longicornis is of particular importance vector for more than 30 human or animal pathogens" should be revised to "H. longicornis is a particularly important vector for more than 30 human and animal pathogens" or "is of particular importance as a vector". In the Results section, "compared to those feds on rabbits" should be "fed on rabbits". Also, "saliva glands" should be "salivary glands". Second, some terms are used inconsistently or incorrectly. The word "alternations" appears multiple times (e.g., "significant alternations") where "alterations" is intended. The phrase "cross alterations" might be better expressed as "reciprocal changes" or "bidirectional remodeling" for clarity. Third, there are minor formatting and punctuation issues, such as missing spaces ("fastp (version0.14.1)", Line 119) and inconsistent use of italics for genus and species names. I recommend that the authors carefully proofread the entire manuscript or seek professional language editing to correct these issues and improve overall readability. Addressing these points will enhance the professional quality of the paper.

Reviewer #2 (Comments for the Author):

This study systematically analyzed the dynamic changes in the microbiome of Haemaphysalis longicornis at different developmental stages (larva, nymph, adult) after blood-feeding on different hosts (mouse vs. rabbit), as well as changes in the host skin microbiome at the bite site. The study revealed vertical transmission of Coxiella and enrichment of Staphylococcus at the bite site.
Specific Comments and Suggestions for Revision
1) In the abstract, the sentence "Overall, microbiome alterations in both tick and host skin varied depending on host species and tick developmental stage" partially repeats the title and the "Importance" section, and does not highlight the core mechanistic findings of the study (e.g., the functional implications of Staphylococcus and Coxiella).
2) It is recommended to add one sentence after the ethics statement: "All animal procedures were performed in accordance with the ARRIVE guidelines and approved by the local ethics committee."
3) The description of the "bite site" sampling method for host skin is not precise enough. Specify whether sterile swabs were used, and whether the swabbing area and number of strokes were standardized. These factors may influence the microbiome results.
4) In the bioinformatics analysis, it is recommended to supplement the chimera removal steps (e.g., VSEARCH, UCHIME), clustering method (e.g., UPARSE, DADA2), similarity threshold (e.g., 97%), and the name and version of the taxonomic

database.
5) In Figures 7A-7D, the PCoA shows that the skin microbiome of bitten mice overlaps more with the tick microbiome than that of rabbits. It is recommended to add PERMANOVA results (R^2, p-value) to quantitatively support the conclusion of "greater overlap."
6) In the Discussion, the authors speculate that differences in host skin sites (back vs. ear) may contribute to the observed host differences, but no literature or experimental support is provided for this speculation. If no data support this, it is recommended to tone down this speculation or explicitly state it as a limitation.
7) In Figure 3A-D (LEfSe), the colors of the icons should be standardized, using the same color for the same annotation. Also, the Venn diagrams in E-F have the same issue; color identification should be unified.
8) The legend of Figure 5B contains an error; it should say "rabbit."
9) Figure 5C-D have no y-axis labels.
10) Figure 1D-F have no y-axis labels.

This study provides a comprehensive and well executed analysis of the stage dependent and host dependent remodeling of both tick and host skin microbiomes during feeding of *Haemaphysalis longicornis*. The authors present compelling evidence that host species and tick developmental stages shape microbial communities, and they highlight the stable vertical transmission of Coxiella and the enrichment of Staphylococcus at bite sites. These findings offer valuable insights into tick host microbial interactions and potential targets for biological control. However, the manuscript would benefit from several improvements.

Comments:

1. The authors have convincingly demonstrated that the microbiome of *Haemaphysalis longicornis* differs significantly between ticks fed on mice versus those fed on rabbits. However, the manuscript does not explore the potential reasons underlying these host dependent differences. I recommend that the authors provide a more in-depth discussion of possible causal factors, such as variations in host blood composition (e.g., nutrients, antibodies, antimicrobial peptides), differential immune pressures exerted by mice and rabbits, or the acquisition of distinct skin associated bacteria during feeding. Addressing these possibilities would strengthen the mechanistic interpretation of the findings and enhance the biological significance of the study.

2. In the Materials and Methods section, the 16S rRNA amplification targets the V3 V4 region, the primer sequences (forward and reverse) are not provided. It would be better to include this information.

3. Figures 2A C and 6A D (LEfSe cladograms). These circular cladograms contain dense taxonomic labels, but the font size and resolution are too low to be legible across all seven panels. No supplementary table lists the differentially abundant taxa (LDA > 2.0, $p < 0.05$) with their enrichment directions. I recommend providing enlarged, high resolution versions as supplementary figures or, preferably, a summary table that clearly presents all significant taxa for each comparison, including taxonomic level, LDA value, p value, and enrichment group.

4. Figures 2E and 2H (nymph stage), and also relevant to 2D 2G and 2F 2I. The Random Forest analysis (Figure 2E) lists the top 15 bacterial genera that discriminate M fed from R fed nymphs, yet only a subset of these (e.g., Coxiella, Cutibacterium, Burkholderia Caballeronia Paraburkholderia) are shown in the relative abundance plot (Figure 2H) with statistical significance indicators. The remaining 12 genera are not presented in Figure 2H, and neither the legend nor the main text explains whether they failed to reach significance in univariate tests. This creates ambiguity for readers who might assume that all top ranked genera from the multivariate model show significant abundance differences. I recommend that the authors clarify in the figure legend or Results section that only those genera with both high importance in Random Forest and significant univariate differences ($p < 0.05$) are displayed in Figures 2G, 2H, and 2I. A brief statement, for example "Among the top 15 genera identified by Random Forest, those with significant relative abundance differences ($p < 0.05$) are shown in G I", would greatly improve transparency.

5. In the Discussion section, the authors frequently restate detailed findings from the Results, which reduces clarity and impact. I suggest substantially condensing this redundancy and instead focusing on two aspects: first, interpreting the biological and ecological implications of the findings; second, addressing the practical significance more clearly. Specifically, at each stage of tick-host interaction, the tick microbiome and host skin microbiome influence each other during coevolution. Understanding this mutual influence can help identify potential measures to protect hosts, including developing new control strategies, drugs, or vaccines. A clearer discussion of this application value would help readers appreciate the study's relevance to the biological control of ticks and tick-borne diseases.

6. The manuscript presents interesting findings, but the writing requires revision to improve clarity, grammar, and professional tone. Several issues were noted throughout the text. First, there are grammatical errors and incorrect word choices. For example, in the Introduction: "H. longicornis is of particular importance vector for more than 30 human or animal pathogens" should be revised to "H. longicornis is a particularly important vector for more than 30 human and animal pathogens" or "is of particular

importance as a vector". In the Results section, "compared to those feds on rabbits" should be "fed on rabbits". Also, "saliva glands" should be "salivary glands". Second, some terms are used inconsistently or incorrectly. The word "alternations" appears multiple times (e.g., "significant alternations") where "alterations" is intended. The phrase "cross alterations" might be better expressed as "reciprocal changes" or "bidirectional remodeling" for clarity. Third, there are minor formatting and punctuation issues, such as missing spaces ("fastp (version0.14.1)", Line 119) and inconsistent use of italics for genus and species names. I recommend that the authors carefully proofread the entire manuscript or seek professional language editing to correct these issues and improve overall readability. Addressing these points will enhance the professional quality of the paper.

Author's Response to Comments

Dear Editor and Reviewers:

Thank you for your professional comments and valuable suggestions concerning our manuscript entitled "**Stage- and host-dependent microbiome remodeling and reciprocal changes between *Haemaphysalis longicornis* and host skin**" (Manuscript ID: Spectrum00864-26). Those comments are very meaningful and very helpful for revising and improving our paper, as well as the important guiding significance to our researches. We have studied comments carefully and have made correction which we hope meet with approval. Revised portion are marked **in red** in our revised manuscript. The main corrections and the responds to all the comments are as following:

Reviewer: 1

This study provides a comprehensive and well executed analysis of the stage dependent and host dependent remodeling of both tick and host skin microbiomes during feeding of *Haemaphysalis longicornis*. The authors present compelling evidence that host species and tick developmental stages shape microbial communities, and they highlight the stable vertical transmission of Coxiella and the enrichment of Staphylococcus at bite sites. These findings offer valuable insights into tick host microbial interactions and potential targets for biological control. However, the manuscript would benefit from several improvements.

Comments:

1. The authors have convincingly demonstrated that the microbiome of *Haemaphysalis longicornis* differs significantly between ticks fed on mice versus those fed on rabbits. However, the manuscript does not explore the potential reasons underlying these host dependent differences. I recommend that the authors provide a more in-depth discussion of possible causal factors, such as variations in host blood composition (e.g., nutrients, antibodies, antimicrobial peptides), differential immune pressures exerted by mice and rabbits, or the acquisition of distinct skin associated bacteria during feeding. Addressing these possibilities would strengthen the mechanistic interpretation of the findings and enhance the biological significance of the study.

Response: We sincerely thank the reviewer for this insightful and constructive comment. According to your kind suggestion, we expanded the Discussion section to address several possible factors that may contribute to these differences. The contents "Consistent with previous studies showing that host blood meal source can substantially alter tick microbial community structure [24-26], our findings further suggest that host-dependent microbiome modulation is a dynamic process throughout tick development. Several mechanisms may contribute to these host-specific effects. Differences in blood nutrient composition, immune factors, and antimicrobial peptides (AMPs), and other bioactive molecules among

vertebrate hosts may selectively change microbial survival and proliferation of specific microorganisms within ticks. In addition, host species differ markedly in the immune responses to tick feeding, which may indirectly alter the tick-associated microbial colonization dynamics. Moreover, ticks are exposed to host skin-associated microorganisms during attachment and blood feeding, providing opportunities for microbial acquisition and exchange [27-30]. Given that the skin microbiota differs substantially between mice and rabbits, exposure to distinct host skin may contribute to the differences in the microbiome observed in ticks feeding on these two host species. Together, the significant microbiome differences observed in our present study maybe driven by a combination of host blood characteristics, host immune-mediated selective pressure, and interactions with host skin-associated microbiota. These findings highlight the complex ecological relationships among ticks, vertebrate hosts, and their associated microbial communities. Nevertheless, the relative contributions of these mechanisms remain unclear and require further experimental validation." have been added in lines 357-377 in our revised manuscript.

2. In the Materials and Methods section, the 16S rRNA amplification targets the V3 V4 region, the primer sequences (forward and reverse) are not provided. It would be better to include this information.

Response: We thank for the reviewer's careful review and valuable suggestion. According to your kind suggestion, we have added the primer

sequence in lines 136-139 in our revised manuscript.

3. Figures 2A C and 6A D (LEfSe cladograms). These circular cladograms contain dense taxonomic labels, but the font size and resolution are too low to be legible across all seven panels. No supplementary table lists the differentially abundant taxa (LDA > 2.0, p < 0.05) with their enrichment directions. I recommend providing enlarged, high resolution versions as supplementary figures or, preferably, a summary table that clearly presents all significant taxa for each comparison, including taxonomic level, LDA value, p value, and enrichment group.

Response: We thank you for your valuable suggestion. To address this concern and improve the presentation of the LEfSe results, we have added the corresponding LDA score (log10) histograms for each comparison as supplementary figures (Figure S2 and Figure S3) in the revised manuscript. These histograms clearly present the significantly differentially abundant taxa identified by LEfSe analysis and their enrichment directions, providing a more accessible and interpretable summary of the results than the cladograms alone. The responding figure legends also have been added in lines 7-13 in supplemental file.

4. Figures 2E and 2H (nymph stage), and also relevant to 2D 2G and 2F 2I. The Random Forest analysis (Figure 2E) lists the top 15 bacterial genera that discriminate M fed from R fed nymphs, yet only a subset of these (e.g., *Coxiella, Cutibacterium, Burkholderia Caballeronia Paraburkholderia*)

are shown in the relative abundance plot (Figure 2H) with statistical significance indicators. The remaining 12 genera are not presented in Figure 2H, and neither the legend nor the main text explains whether they failed to reach significance in univariate tests. This creates ambiguity for readers who might assume that all top ranked genera from the multivariate model show significant abundance differences. I recommend that the authors clarify in the figure legend or Results section that only those genera with both high importance in Random Forest and significant univariate differences ($p < 0.05$) are displayed in Figures 2G, 2H, and 2I. A brief statement, for example "Among the top 15 genera identified by Random Forest, those with significant relative abundance differences ($p < 0.05$) are shown in G I", would greatly improve transparency.

Response: We agree and thanks for the reviewer's valuable suggestion. To improve clarity and transparency, we have revised both the Results section and the figure legends for Figures 2G-2I. The contents "Among these genera identified by Random Forest, those with significant differences in relative abundance ($p < 0.05$) were shown in Figure 2G-2I." have been added in lines 218-220 (section Results), and the contents "Relative abundance plots of bacterial genera among the top predictors identified by Random Forest analysis that exhibited significant differences in relative abundance ($p < 0.05$)" have been added in lines 584-586 (section Figure legends) in our revised manuscript.

5. In the Discussion section, the authors frequently restate detailed findings from the Results, which reduces clarity and impact. I suggest substantially condensing this redundancy and instead focusing on two aspects: first, interpreting the biological and ecological implications of the findings; second, addressing the practical significance more clearly. Specifically, at each stage of tick-host interaction, the tick microbiome and host skin microbiome influence each other during coevolution. Understanding this mutual influence can help identify potential measures to protect hosts, including developing new control strategies, drugs, or vaccines. A clearer discussion of this application value would help readers appreciate the study's relevance to the biological control of ticks and tick-borne diseases.

Response: We sincerely thank the reviewer for the constructive suggestion. In response, we have substantially revised the Discussion section by reducing detailed restatements of the experimental findings instead focusing on two aspects: (I) the biological and ecological implications of the observed host- and stage-dependent microbiome interactions, and (II) their potential practical significance. We highlight that these reciprocal interactions may influence tick adaptation, host responses, and potentially pathogen transmission dynamics. Also, we strengthened the section addressing the applied relevance of our findings, discussing how microbiome-associated mechanisms may provide new opportunities for

ticks and tick-borne disease control strategies. The revised parts in Discussion part (lines 355-382, 390-398, 409-414) have been marked in red in our revised manuscript.

6. The manuscript presents interesting findings, but the writing requires revision to improve clarity, grammar, and professional tone. Several issues were noted throughout the text. First, there are grammatical errors and incorrect word choices. For example, in the Introduction: "H. longicornis is of particular importance vector for more than 30 human or animal pathogens" should be revised to "H. longicornis is a particularly important vector for more than 30 human and animal pathogens" or "is of particular importance as a vector". In the Results section, "compared to those feds on rabbits" should be "fed on rabbits". Also, "saliva glands" should be "salivary glands". Second, some terms are used inconsistently or incorrectly. The word "alternations" appears multiple times (e.g., "significant alternations") where "alterations" is intended. The phrase "cross alterations" might be better expressed as "reciprocal changes" or "bidirectional remodeling" for clarity. Third, there are minor formatting and punctuation issues, such as missing spaces ("fastp (version0.14.1)", Line 119) and inconsistent use of italics for genus and species names. I recommend that the authors carefully proofread the entire manuscript or seek professional language editing to correct these issues and improve overall readability. Addressing these points will enhance the professional

quality of the paper.

Response: We sincerely thank the reviewer for the careful evaluation of our manuscript and for the constructive comments regarding language, terminology, and formatting. In accordance with these valuable suggestions, we have carefully reviewed the entire manuscript and thoroughly revised the text. To further improve the language quality, we have carefully revised the manuscript with the assistance of native English-speaking colleagues and professional language-editing service (LetPub).

All corresponding revisions have been highlighted in red in the revised manuscript. We hope that these revisions adequately address the reviewer's concerns and improve the clarity, readability, and overall quality of the manuscript. We greatly appreciate the reviewer's careful review and valuable suggestions, which have helped us substantially improve the manuscript.

According to the reviewers' valuable suggestions, we have carefully revised all the issues raised. The revised sections are highlighted in red in the revised manuscript. We sincerely thank the reviewer again for your careful evaluation and constructive suggestions, which have significantly improved the quality, clarity, and overall presentation of our work.

Reviewer: 2

This study systematically analyzed the dynamic changes in the microbiome of *Haemaphysalis longicornis* at different developmental stages (larva, nymph, adult) after blood-feeding on different hosts (mouse vs. rabbit), as well as changes in the host skin microbiome at the bite site. The study revealed vertical transmission of *Coxiella* and enrichment of *Staphylococcus* at the bite site.

Specific Comments and Suggestions for Revision.

1. In the abstract, the sentence "Overall, microbiome alterations in both tick and host skin varied depending on host species and tick developmental stage" partially repeats the title and the "Importance" section, and does not highlight the core mechanistic findings of the study (e.g., the functional implications of *Staphylococcus* and *Coxiella*)

Response: We thank the reviewer for the valuable suggestion. Accordingly, we have revised the concluding part of the Abstract to better emphasize the major findings of our study. The original contents have been changed to "Notably, *Coxiella* was identified as the dominant and stable bacterial symbiont across developmental stages and tissues, and was consistently detected in eggs, suggesting that persistent vertical transmission may contribute to tick fitness development, and nutritional homeostasis. On the other hand, *Staphylococcus* was identified to be consistent enrichment at tick bite sites across host species and developmental stages, indicating that it may represent a key microbial responder involved in local microbiome

remodeling and host skin responses to tick feeding. Collectively, these findings suggest that blood feeding drives dynamic remodeling of both tick and host skin-associated microbiomes, and highlight *Coxiella* persistence and *Staphylococcus* enrichment as key microbial signatures of tick-host microbial interactions. Our study advances current understanding of microbiome cross-talk at the tick-host interface and provides new insights into microbiome-mediated mechanisms that may influence tick adaptation, host responses, and pathogen transmission. These findings also highlight potential opportunities for the development of microbiome-based strategies for the control of ticks and tick-borne diseases." in lines 28-42 in our revised manuscript.

2. It is recommended to add one sentence after the ethics statement: "All animal procedures were performed in accordance with the ARRIVE guidelines and approved by the local ethics committee.".

Response: According to your kind suggestion, we have added the contents "All animal procedures were performed in accordance with the ARRIVE guidelines and approved by the local ethics committee" in lines 100-102 in our revised manuscript.

3. The description of the "bite site" sampling method for host skin is not precise enough. Specify whether sterile swabs were used, and whether the swabbing area and number of strokes were standardized. These factors may influence the microbiome results.

Response: We thank the reviewer for the valuable comment. To address

this concern, we have added the details about sampling method for host skin in the Materials and Methods section. Specifically, sterile swabs were used for all skin microbiome sampling. For each sample, the skin was swabbed using a standardized sampling area surrounding the tick bite site (approximately 1.5 cm × 1.5 cm) and 6 swabbing strokes while applying consistent pressure. All samples were collected by the same investigator following the same procedure to minimize technical variation among groups. According to your valuable suggestion, we have revised the responding description, the original contents have been changed to "For skin microbiome sampling, skin swabs were collected from mice and rabbits at tick bite sites after all ticks had detached. Samples were obtained from the skin area enclosed by the feeding capsule in mice or the ear bag in rabbits, thereby ensuring a standardized sampling area (approximately 1.5 cm × 1.5 cm) corresponding to the region exposed to tick attachment and blood-feeding. The control samples from mice or rabbit that were not infested with ticks but under the same experimental procedures. All samples were collected using sterile PBS-moistened swabs. To minimize technical variation, all sampling procedures were performed by the same investigator following an identical sampling protocol throughout the study" in lines 122-130 in our revised manuscript.

4. In the bioinformatics analysis, it is recommended to supplement the chimera removal steps (e.g., VSEARCH, UCHIME), clustering method (e.g., UPARSE, DADA2), similarity threshold (e.g., 97%), and the name

and version of the taxonomic database.

Response: According to the reviewer's kind suggestion, the contents "Tags were clustered with a 100% sequence similarity for 16S rRNA genes using DADA2_QIIME2, and chimeric sequences were removed using the DADA2_QIIME2. Taxonomic annotation of sequences was performed with different annotation databases, named Silva database (v.132 SSURef)." have been added in lines 147-150 in our revised manuscript.

5. In Figures 7A-7D, the PCoA shows that the skin microbiome of bitten mice overlaps more with the tick microbiome than that of rabbits. It is recommended to add PERMANOVA results (R^2, p-value) to quantitatively support the conclusion of "greater overlap."

Response: We sincerely appreciate the reviewer for the professional comment and valuable suggestion. Based on the reviewer's kind suggestion, we performed additional PERMANOVA analyses to provide quantitative support for the observed clustering patterns and included the corresponding R^2 and p values in our revised manuscript. The original responding description have been changed to "The results showed that the microbiome of tick-bitten mice was more similar to the tick microbiome ($R^2 = 0.341$, $p = 0.001$) than that of control mice ($R^2 = 0.686$, $p = 0.001$). In rabbits, the microbiome of tick-bitten skin showed comparable levels of dissimilarity to both the tick microbiome ($R^2 = 0.250$, $p = 0.001$) and the control rabbit skin microbiome ($R^2 = 0.233$, $p = 0.006$), indicating a less pronounced microbial shift following tick feeding." in lines 328-334 in our

revised manuscript.

6. In the Discussion, the authors speculate that differences in host skin sites (back vs. ear) may contribute to the observed host differences, but no literature or experimental support is provided for this speculation. If no data support this, it is recommended to tone down this speculation or explicitly state it as a limitation.

Response: We sincerely appreciate the reviewer's insightful comments and constructive suggestions. We agree that our previous discussion regarding the potential influence of different skin sites on microbiome composition was speculative and was not directly supported by data. To avoid overinterpretation, we have removed this statement from the Discussion section. According to your valuable suggestion, the original contents have been changed to "After tick feeding, the skin-associated changes in mice predominantly involved key bacterial genera including *Staphylococcus*, *Pseudomonas*, and *Kocuria*, whereas in rabbits, the major changes were associated with *Staphylococcus*, *Pseudomonas*, and *Streptococcus*. The underlying mechanisms driving these host-specific and stage-dependent effects remain unclear and warrant further investigation." in lines 390-395 in our revised manuscript.

7. In Figure 6A-D (LEfSe), the colors of the icons should be standardized, using the same color for the same annotation. Also, the Venn diagrams in E-F have the same issue; color identification should be unified.

Response: According to your kind advice, we have revised the color

scheme across the LEfSe panels (Figure 6A-D), ensuring that the same group is represented by the same color throughout. In addition, the color identification in the Venn diagrams (Figure 6E-F) has also been revised accordingly. The revised figures are presented in our updated manuscript.

8. The legend of Figure 5B contains an error; it should say "rabbit."

Response: We sincerely thank the reviewer for carefully pointing out this error. We are sorry for our careless mistake in the legend of Figure 5B, we have corrected it in our revised manuscript (lines 610).

9. Figure 5C-D have no y-axis labels

Response: According to the reviewer's kind advice, we have updated Figure 5C-D by adding y-axis labels to improve figure clarity.

10. Figure 1D-F have no y-axis labels.

Response: Thank you for pointing out this issue. We have revised Figure 1D-F by adding the appropriate y-axis labels to each panel in our revised manuscript.

According to the reviewers' valuable advice, all the issues have been carefully revised. Revised portion are marked **in red** in our revised manuscript. We sincerely thank the reviewer again for the careful evaluation and the valuable suggestions. These constructive comments have been highly beneficial in improving the quality, clarity, and overall presentation of our work.

Re: Spectrum00864-26R1 (Stage- and host-dependent microbiome remodeling and reciprocal changes between Haemaphysalis longicornis and host skin)

Dear Dr. Zhibing Lin:

Thank you for the privilege of reviewing your work. Below you will find my comments, instructions from the Spectrum editorial office, and the reviewer comments.

Both reviewers recognize the value and significance of the study; however, the methods section requires further revision.

Please return the manuscript within 60 days; if you cannot complete the modification within this time period, please contact me. If you do not wish to modify the manuscript and prefer to submit it to another journal, notify me immediately so that the manuscript may be formally withdrawn from consideration by Spectrum.

Revision Guidelines
To submit your modified manuscript, log into the submission site at https://spectrum.msubmit.net/cgi-bin/main.plex. Go to Author Tasks and click the appropriate manuscript title to begin. The information you entered when you first submitted the paper will be displayed; update this as necessary. Note the following requirements:

• Upload point-by-point responses to the issues raised by the reviewers in a file named "Response to Reviewers," NOT in your cover letter.
• Upload a compare copy of the manuscript (without figures) as a "Marked-Up Manuscript" file.
• Upload a clean .DOC/.DOCX version of the revised manuscript and remove the previous version.
• Each figure must be uploaded as a separate, editable, high-resolution file (TIFF or EPS preferred), and any multipanel figures must be assembled into one file.
• Any supplemental material intended for posting by ASM should be uploaded with their legends separate from the main manuscript. You can combine all supplemental material into one file (preferred) or split it into a maximum of 10 files with all associated legends included.

For complete guidelines on revision requirements, see our Submission and Review Process webpage. Submission of a paper that does not conform to guidelines may delay acceptance of your manuscript.

Data availability: ASM policy requires that data be available to the public upon online posting of the article, so please verify all links to sequence records, if present, and make sure that each number retrieves the full record of the data. If a new accession number is not linked or a link is broken, provide Spectrum production staff with the correct URL for the record. If the accession numbers for new data are not publicly accessible before the expected online posting of the article, publication may be delayed; please contact production staff (Spectrum@asmusa.org) immediately with the expected release date.

Publication Fees: For information on publication fees and which article types are subject to charges, visit our website. If your manuscript is accepted for publication and any fees apply, you will be contacted separately about payment during the production process; please follow the instructions in that e-mail. Arrangements for payment must be made before your article is published.

ASM Membership: Corresponding authors may join or renew ASM membership to obtain discounts on publication fees. Need to upgrade your membership level? Please contact Customer Service at Service@asmusa.org.

The ASM Journals program strives for constant improvement in our submission and publication process. Please tell us how we can improve your experience by taking this quick Author Survey.

Thank you for submitting your paper to Spectrum.

Sincerely,
Xinzhao Tong
Editor
Microbiology Spectrum

Reviewer #1 (Comments for the Author):

all my concerns have been addressed

Reviewer #2 (Comments for the Author):

1. In the Materials and Methods section, the authors state that "All animal procedures were performed in accordance with the ARRIVE guidelines and approved by the local ethics committee," but this is not explicitly presented in a separate ethics statement section. It is suggested to add an independent "Ethics statement" subsection at the end of the Materials and Methods to list the above declaration separately.
2. The bioinformatics analysis section does not provide specific tools for chimera removal (e.g., VSEARCH, UCHIME), ASV/OTU clustering methods (denoising parameters of DADA2), similarity thresholds, or the full version information of the taxonomic databases.
3. For the LEfSe analysis, it is unclear whether Benjamini-Hochberg FDR correction was applied and what the significance threshold was after correction. For the Random Forest analysis, the specific method of model validation (e.g., number of cross-validation folds, out-of-bag error, etc.) should be provided. For alpha diversity comparisons, the specific statistical test methods used (e.g., Kruskal-Wallis test with Dunn's post-hoc test, or t-test) should be clarified.
4. In Figure 3D (LEfSe analysis), the color coding for each group is inconsistent across different subpanels.
5. There are a few minor language issues in the text, for example: (1) In the abstract (Line 32), "Staphylococcus was identified to be consistent enrichment" is grammatically incorrect and should be "was consistently enriched"; (2) In the PCoA analysis description for Figure 1 (Line 179-180), the authors used "艾at the larval" which appears to be a typographical error.

Author's Response to Comments

Dear Editor and Reviewer:

Thank you for your professional comments and valuable suggestions concerning our manuscript entitled "**Stage- and host-dependent microbiome remodeling and reciprocal changes between *Haemaphysalis longicornis* and host skin**" (Manuscript ID: Spectrum00864-26). Those comments are very meaningful and very helpful for revising and improving our paper, as well as the important guiding significance to our researches. We have studied comments carefully and have made correction which we hope meet with approval. Revised portion are marked **in red** in our revised manuscript. The main corrections and the responds to all the comments are as following:

Reviewer: 2

1. In the Materials and Methods section, the authors state that "All animal procedures were performed in accordance with the ARRIVE guidelines and approved by the local ethics committee," but this is not explicitly presented in a separate ethics statement section. It is suggested to add an independent "Ethics statement" subsection at the end of the Materials and Methods to list the above declaration separately.

Response: We sincerely thank the reviewer for this valuable suggestion. In accordance with the reviewer's recommendation, we have added a separate "Ethics Statement" subsection at the end of the Materials and

Methods section (lines 165-171).

2. The bioinformatics analysis section does not provide specific tools for chimera removal (e.g., VSEARCH, UCHIME), ASV/OTU clustering methods (denoising parameters of DADA2), similarity thresholds, or the full version information of the taxonomic databases.

Response: We sincerely thank the reviewer for this constructive suggestion. Following the reviewer's recommendation, we have further expanded the Bioinformatics Analysis section by providing additional details on the sequence processing workflow, including the software and versions used for read merging, DADA2-based denoising, chimera removal, ASV inference, and taxonomic assignment. The contents "(version 0.14.1)" and "(version 1.14)" have been added in lines 142 and 143, separately. The contents "Based on the overlap between paired-end reads, USEARCH (v 10) -fastq_mergepairs was applied to merge read pairs, discard non-conforming tags, and generate raw tags. Clean tags were imported into QIIME2 and processed using the DADA2 pipeline (q2-dada2, v 2020.11.0) for quality filtering, denoising, and amplicon sequence variant (ASV) inference at single-nucleotide resolution (100% sequence identity). Chimeric sequences were identified and removed using the consensus method implemented in DADA2 (v 2020.11.0). Non-chimeric ASVs were subsequently assigned taxonomy against the silva database (v 132) using the corresponding pre-trained classifier at the default confidence threshold."

have been added in lines 143-151 in our revised manuscript.

3. For the LEfSe analysis, it is unclear whether Benjamini-Hochberg FDR correction was applied and what the significance threshold was after correction. For the Random Forest analysis, the specific method of model validation (e.g., number of cross-validation folds, out-of-bag error, etc.) should be provided. For alpha diversity comparisons, the specific statistical test methods used (e.g., Kruskal-Wallis test with Dunn's post-hoc test, or t-test) should be clarified.

Response: We sincerely thank the reviewer for these constructive comments. Following the reviewer's suggestions, we have revised the Statistical Analysis and Bioinformatics Analysis sections to provide additional methodological details.

① **LEfSe analysis:** According to the reviewer's kind suggestion, the contents "Taxa with an LDA score >2.0 and a permutation-based FDR-adjusted *p* value <0.05 were considered significant discriminative biomarkers." have been added in lines 171-173 in our revised manuscript.

② **Random Forest analysis:** Thank you for the valuable comment. We have revised the Material and methods section to provide additional details regarding the construction and validation strategy of the Random Forest model. The corresponding contents "Random Forest analysis was performed using the Magigene Cloud Platform. The number of decision trees (ntree) was set to 500. Feature importance was evaluated using Mean Decrease Accuracy (MDA) indices. A random partition-based prediction

strategy was applied for model construction and evaluation." have been added in lines 159-162 in our revised manuscript.

③**Alpha diversity analysis:** The contents "Kruskal-Wallis test followed by a post hoc test (dunn's test) was used when more than two group settings, and *t*-test was used when two groups were compared." have been added in lines 173-175 in our revised manuscript.

4. -In Figure 3D (LEfSe analysis), the color coding for each group is inconsistent across different subpanels.

Response: Thank you for the reviewer's helpful comment. We have revised Figure 3D to improve the consistency and clarity of the color coding. Specifically, the order of the group labels in the figure legend has been adjusted to correspond to the order of the colored bars in the LEfSe histogram, making the color assignments easier to follow.

5. There are a few minor language issues in the text, for example: (1) In the abstract (Line 32), "*Staphylococcus* was identified to be consistent enrichment" is grammatically incorrect and should be "was consistently enriched"; (2) In the PCoA analysis description for Figure 1 (Line 179-180), the authors used "艾at the larval" which appears to be a typographical error.

Response: Thank you for your careful reading of our manuscript and for pointing out these language issues. We sincerely apologize for these oversights. We have corrected the grammatical error in the Abstract. In addition, the typographical error "艾 at the larval" has been corrected to

“at the larval.” Furthermore, we carefully checked the entire manuscript and corrected minor grammatical and typographical errors to improve the overall clarity and readability.

According to the reviewers’ valuable advice, all the issues have been carefully revised. Revised portion are marked **in red** in our revised manuscript. We sincerely thank the reviewer again for the careful evaluation and the valuable suggestions. These constructive comments have been highly beneficial in improving the quality, clarity, and overall presentation of our work.

Re: Spectrum00864-26R2 (Stage- and host-dependent microbiome remodeling and reciprocal changes between Haemaphysalis longicornis and host skin)

Dear Dr. Zhibing Lin:

After careful evaluation by the reviewers, all concerns raised previously have been addressed.

Your manuscript has been accepted, and I am forwarding it to the ASM production staff for publication. Your paper will first be checked to make sure all elements meet the technical requirements. ASM staff will contact you if anything needs to be revised before copyediting and production can begin. Otherwise, you will be notified when your proofs are ready to be viewed.

Data Availability: ASM policy requires that data be available to the public upon online posting of the article, so please verify all links to sequence records, if present, and make sure that each number retrieves the full record of the data. If a new accession number is not linked or a link is broken, provide production staff with the correct URL for the record. If the accession numbers for new data are not publicly accessible before the expected online posting of the article, publication may be delayed; please contact ASM production staff immediately with the expected release date.

Publication Fees: For information on publication fees and which article types have charges, please visit ourwebsite. We have partnered with Copyright Clearance Center (CCC) to collect author charges. If fees apply to your paper, you will receive a message from no-reply@copyright.com with further instructions. For questions related to paying charges through RightsLink, please contact CCC at ASM_Support@copyright.com or toll free at +1-877-622-5543. CCC makes every attempt to respond to all emails within 24 hours.

ASM Membership: Corresponding authors may join or renew ASM membership to obtain discounts on publication fees. Need to upgrade your membership level? Please contact Customer Service at Service@asmusa.org.

Embargo Policy: A press release may be issued as soon as the manuscript is posted on theSpectrum Latest Articles webpage. The corresponding author will receive an email with the subject line "ASM Journals Author Services Notification" when the article is available online.

Cover Image Submissions: If you would like to submit a potential Cover Image, please email a file and a short legend to spectrum@asmusa.org. Please note that we can only consider images that (i) the authors created or own and (ii) have not been previously published. By submitting, you agree that the image can be used under the same terms as the published article. Image File requirements: TIF/EPS, 7.5 inches wide by 8.25 inches tall (at least 2,250 pixels wide by 2,475 pixels tall), minimum 300 dpi resolution (600 dpi preferred), RGB, and no figure elements, e.g., arrows or panel labels. The legend should be a short description of the image, 1-2 sentences recommended. Please download and use this interactive template in Adobe the https://journals.asm.org/pb-assets/html/asm-cover-sizing-tool.html to ensure that your proposed cover image meets our size requirements (https://journals.asm.org/pb-assets/pdf-text-excel-files/ASM-Interactive-Sizing-Cover-Template-1715689791.pdf).

The ASM Journals program strives for constant improvement in our submission and publication process. Please tell us how we can improve your experience by taking this quick Author Survey.

Thank you for submitting your paper to Spectrum.

Sincerely,
Xinzhao Tong
Editor
Microbiology Spectrum

Reviewer #2 (Comments for the Author):

All my concerns have been addressed.
