# Supplementary material for: Stage- and host-dependent microbiome remodeling and reciprocal changes between Haemaphysalis longicornis and host skin
Source: Microbiol Spectr. 2026 Aug 21;14(10):e00864-26. doi: 10.1128/spectrum.00864-26 (PMC13689275; doi:10.1128/spectrum.00864-26)
Supplement: Supplementary file 2 [file spectrum.00864-26-s0001.docx]

**Figure S1** **Differential alterations in bacterial microbial diversity of ticks at different developmental stages following blood-feeding on different hosts. (A-C)** Average compositions and relative abundances of Top 20 most abundant phyla for fed larva **(A)**, nymph **(B)** and adult **(C)**. **(D-F)** Venn diagram showing the shared and unique ASVs between M-fed larva and R-fed larva **(D)**, between M-fed nymph and R-fed nymph **(E)**, between M-fed adult and R-fed adult **(F)**.

**Figure S2 LEfSe analysis between different groups.** LDA score histograms (LDA score > 2.0) showing differential taxa between M-fed and R-fed ticks at larval (A), nymphal (B), and adult (C) stages.

**Figure S3 LEfSe analysis among different groups.** LDA score histograms showing differentially abundant taxa (LDA score > 2.0) among control and tick-bitten mice (A), mice bitten by ticks at different developmental stages (B), and rabbits bitten by ticks at different developmental stages (C).
